# Engineered microbial biofuel production and recovery under supercritical carbon dioxide

**DOI:** 10.1038/s41467-019-08486-6

**Published:** 2019-02-04

**Authors:** Jason T. Boock, Adam J. E. Freedman, Geoffrey A. Tompsett, Sarah K. Muse, Audrey J. Allen, Luke A. Jackson, Bernardo Castro-Dominguez, Michael T. Timko, Kristala L. J. Prather, Janelle R. Thompson

**Affiliations:** 1 Department of Chemical Engineering, Massachusetts Institute of Technology, Cambridge, MA 02139 USA; 2 Department of Civil and Environmental Engineering, Massachusetts Institute of Technology, Cambridge, MA 02139 USA; 3 Department of Chemical Engineering, Worcester Polytechnic Institute, Worcester, MA 01609 USA; 4 Department of Chemical Engineering, University of Bath, Bath, BA2 7AY UK; 5 Present Address: Department of Chemical, Paper, and Biomedical Engineering, Miami University, Oxford, OH 45056 USA

## Abstract

Culture contamination, end-product toxicity, and energy efficient product recovery are long-standing bioprocess challenges. To solve these problems, we propose a high-pressure fermentation strategy, coupled with in situ extraction using the abundant and renewable solvent supercritical carbon dioxide (scCO_2_), which is also known for its broad microbial lethality. Towards this goal, we report the domestication and engineering of a scCO_2_-tolerant strain of *Bacillus megaterium*, previously isolated from formation waters from the McElmo Dome CO_2_ field, to produce branched alcohols that have potential use as biofuels. After establishing induced-expression under scCO_2_, isobutanol production from 2-ketoisovalerate is observed with greater than 40% yield with co-produced isopentanol. Finally, we present a process model to compare the energy required for our process to other in situ extraction methods, such as gas stripping, finding scCO_2_ extraction to be potentially competitive, if not superior.

## Abstract (web summary)

End-product toxicity, culture contamination, and energy efficient product recovery are long-standing issues in bioprocessing. Here, the authors address these problems using a fermentation strategy that combines microbial production of branched alcohols with supercritical carbon dioxide extraction.

## Introduction

Supercritical fluids offer many advantages over conventional solvents due to their gas-like viscosity and diffusivity with liquid-like density and solvation^1^. Supercritical carbon dioxide (scCO_2_) is especially promising as a green solvent due to its moderate critical point temperature (31.1 °C) and pressure (7.38 MPa) as well as being non-flammable, non-hazardous, abundant, and inexpensive^2^. Numerous biocatalytic processes have taken advantage of scCO_2_^2,3^, including enantiomer specific lipase reactions^4^, asymmetric alcohol reduction^5^, and carboxylation of various substrates^6^. The solvent properties of scCO_2_ have been exploited for the separation of alcohols, aldehydes, ketones, and acids from aqueous solutions via both in situ and ex situ extraction^7^. In situ product extraction has been shown to have a dramatic effect on bioproduction titers by alleviating end-product inhibition or toxicity^8–11^. scCO_2_ is especially attractive within biphasic scCO_2_-aqueous systems^12^ due to its preferential extraction of alcohols with intermediate-chain length (e.g., 3 to 6 carbons), the result of decreasing water solubility with increasing number of carbons. Conversely, alcohol volatility decreases with chain length, making these molecules more attractive targets for scCO_2_ extraction compared to gas stripping^12^.

Despite the potential to couple scCO_2_ for product removal with microbial synthesis of target compounds, the two have not been combined due to the broad microbial lethality of scCO_2_^13^, where cellular inactivation is thought to occur through a combination of membrane disruption, cytosolic acidification, enzyme inactivation, and cellular desiccation^14^. Several early studies have explored processes catalyzed by whole cells under scCO_2_; however, these were conducted over short time scales ( < 1 day) using biomass grown in the absence of scCO_2_, and without evidence of cell viability under scCO_2_. Two results include carboxylation of pyrrole substrate with *Bacillus megaterium* cells in a biphasic mixture of buffered solution and scCO_2_ after 1 h^15^, and reduction of ketones with a high degree of enantiomer specificity using immobilized *Geotrichum candidum* cells in an aqueous-scCO_2_ biphasic continuous flow reactor^16^. Several additional studies have employed scCO_2_-based extraction of bioproducts, but have not achieved in situ extraction due to inhibited cell growth. Using a semi-continuous process, ethanol was extracted from the spent fermentation broth of yeast^17^ and *Clostridium*^18^ cultures using scCO_2_. Knutsen et al.^19^ attempted to produce ethanol from cellobiose in dual-phase aqueous bioreactors containing non-growing *Clostridium thermocellum* and pressurized scCO_2_, N_2_ or ethane headspaces. Significant cellobiose conversion and ethanol production were observed under pressurized nitrogen and ethane, but not under scCO_2_^19^, indicating process inhibition by CO_2_.

Bioprospecting is a promising approach to recover organisms capable of active metabolism under high pressures of CO_2_. Numerous environmental isolates have been characterized with tolerance to high partial pressures of CO_2_^20^, although rarely are cultures examined above atmospheric pressures (0.1 MPa). Santillan et al.^21^ found a strain of *Lactobacillus casei* in a terrestrial CO_2_-rich spring that can grow in CO_2_ pressures up to 1 MPa. Recently, Peet et al.^22^ examined organisms capable of growth at geologic CO_2_ sequestration sites, isolating several endospore-forming *Bacillus* species that are tolerant to scCO_2_ treatment ( > 10 MPa), but with low growth frequency and magnitude. Similarly, growth was reported for laboratory cultures inoculated with deep subsurface sandstone under scCO_2_, although the identities of microorganisms were not determined^23^. Motivated by the hypothesis that natural deposits of high-pressure CO_2_ would harbor microorganisms adapted to actively grow in close contact with it, Freedman et al.^24^ investigated the microbial diversity at McElmo Dome, a deep subsurface CO_2_ reservoir that was formed 40–72 million years ago. Laboratory enrichment cultivation of fluids collected from McElmo dome under scCO_2_ enabled isolation of *B. megaterium* strain SR7, an endospore-forming, facultative-anaerobe^25^. We have observed growth of SR7 cultures to a cell density of greater than 10^7^ cells ml^–1^ after 3 weeks under scCO_2_ following inoculation as endospores and supplementation with the germination inducer l-alanine^25^. Endospores of Gram-positive bacteria are known for their resiliency even when exposed directly to scCO_2_^22,26^, and we have hypothesized that this dormant state enables these bacteria to withstand and adapt to the scCO_2_ environment prior to outgrowth as vegetative cells. Analysis of fermentation products from SR7 grown under scCO_2_ indicated the production of lactate, acetate and succinate, demonstrating active metabolism under scCO_2_ and the potential for microbial production to be coupled with scCO_2_ extraction^25^.

**Fig. 1:**
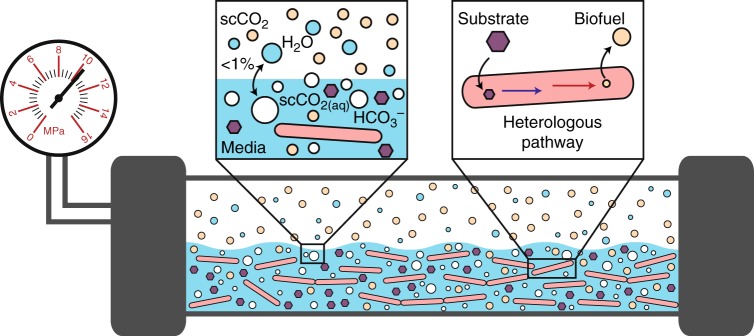
Schematic of integrated fermentation and extraction under supercritical CO_2_. A scCO_2_-tolerant microbe can be engineered to produce compounds, such as medium-chain alcohols (C4-C5), that may serve as biofuels and may be preferentially extracted from aqueous media into the scCO_2_ phase. Collection of scCO_2_ followed by partial de-pressurization will facilitate high-purity biofuel extraction. The presented biphasic scCO_2_ separation strategy is expected to simultaneously provide a contaminant-free environment for the engineered organism due to the broad microbial lethality of scCO_2_, and continuously strip microbially produced biofuels to eliminate end-product toxicity. scCO_2_ is only weakly soluble in the aqueous media phase ( < 1%) and vice versa, resulting in a biphasic system

In this work, we seek to develop *B. megaterium* SR7 as a bioproduction host under scCO_2_ for useful compounds that have already been established for in situ extraction using scCO_2_ (Fig. 1), such as branched, intermediate-chain alcohols. We posit that an integrated fermentation-scCO_2_ extraction process may ultimately, and simultaneously, solve three long-standing challenges in the field by (1) reducing end-product toxicity through extraction^9–11^, (2) mitigating culture contamination under the highly selective conditions of scCO_2_^13,22,25^, and (3) providing an energy efficient method to recover high-purity products using scCO_2_ as a sustainable solvent^1,12^. Branched, intermediate-chain alcohols are not naturally made by SR7, necessitating metabolic engineering to generate them^25^. Isobutanol is selected due to its importance as a drop-in replacement for gasoline and its favorable fuel characteristics (i.e., high energy density, suitable research octane number, low hygroscopicity). Additionally, isobutanol is an attractive molecule for in situ scCO_2_ extraction as it is cytotoxic and expected to partition favorably to the scCO_2_ phase^7,12^. Isobutanol production requires introduction of two heterologous enzymes, an α-ketoisovalerate decarboxylase and alcohol dehydrogenase, to convert the valine-synthesis intermediate, 2-ketoisovalerate, to the final product with minimal intermediate isobutyraldehyde accumulation^27^. We conclude our study by analyzing the energy requirements of an integrated fermentation-extraction process, comparing it to alternative in situ extraction technologies such as gas stripping, pervaporation, and adsorption^28^, in order to address challenges associated with energy efficient product recovery.

## Results

### Heterologous protein expression in aerobically grown SR7

**Fig. 2:**
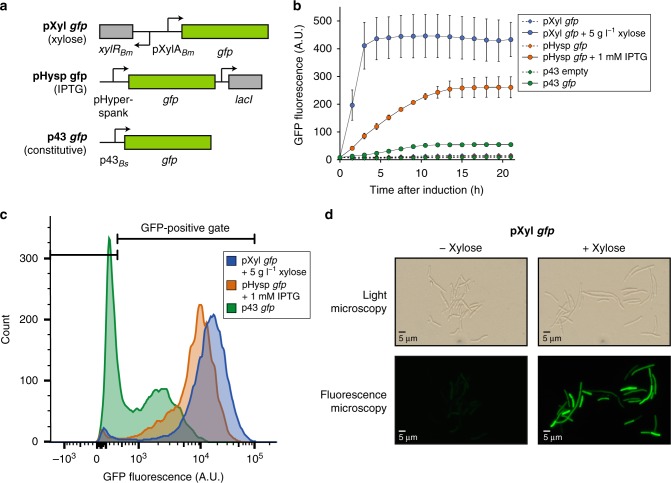
Heterologous protein expression and promoter evaluation in SR7. **a** Schematic of the promoters tested in SR7. pXyl is a xylose-inducible promoter and contains the *xylR* gene and *xylA* promoter from *B. megaterium* strain DSM 319. pHysp contains the IPTG-inducible hyperspank promoter and *lacI*, both from plasmid pDR111. The p43 promoter is taken from *B. subtilis* strain KS438 and is growth-associated. Expected inducers are indicated in parentheses. **b** Bulk fluorescence measurements of SR7 populations containing *gfp-*encoding plasmids, with (circles, solid lines) or without (diamonds, dashed lines) inducers. Error bars represent the standard deviation of biological triplicate samples. **c** Fluorescence populations of SR7 cells expressing *gfp* at 4 h post induction measured by FACS (Alexa Fluor 488). A GFP positive gate was established using the autofluorescence of SR7 cells containing an empty plasmid control. **d** Phase-contrast and fluorescence microscopy of SR7 pXyl *gfp* cells with and without xylose induction. Images were taken 4 h post induction. Source data are provided as a Source Data file

As strain *B. megaterium* SR7 is an environmental isolate, its genetic modification required optimization and customization of protocols previously developed for laboratory strains and model organisms. A transformation protocol for SR7 was developed by modifying a method based on protoplast-osmotic shock^29^ (Supplementary Note 1). The xylose-inducible promoter system (pXyl) was selected for use in SR7 due to its simplicity, high expression level, and widespread use in the *B. megaterium* literature^29^ (Fig. 2a). Additionally, the IPTG-inducible hyperspank promoter system (pHysp)^30^ and the p43-growth based promoter (p43) were evaluated, both of which have shown effective protein production in *B*. *subtilis*^31^ (Fig. 2a).

All three promoters were functional in SR7 (Fig. 2b and Supplementary Fig. 1), with pXyl and pHysp promoters resulting in strong GFP expression of 25- and 20-fold greater than uninduced cultures, respectively. The xylose promoter was activated most rapidly, achieving maximal fluorescence 3 h post induction, similar to other *B. megaterium* strains using this promoter system^32^. For induced pXyl *gfp* and pHysp *gfp* cultures, a large proportion ( > 95%) of the population was found to express GFP (Fig. 2c, d and Supplementary Table 1), which is greater than that observed for other *B. megaterium* strains using pXyl^33^. Uninduced pXyl *gfp* containing cells have many members (40%) that fall into the GFP positive gate, providing evidence that this promoter is leaky when grown aerobically in rich Luria-Bertani (LB) medium (Supplementary Table 1 and Supplementary Fig. 2), in contrast to previous work showing tight regulation of pXyl within *B. megaterium* strain WH320^32^. As expected, the growth-associated p43 promoter was activated as the cells entered exponential growth phase (Supplementary Fig. 1e, f), but showed weaker GFP expression, only sixfold greater than the empty plasmid-containing control strain. Inducible production of GFP for the pXyl and pHysp promoters was also observed for cells cultured in semi-defined medium supplemented with xylose and/or glucose, with maximal fluorescence observed for cells containing the pXyl promoter grown in xylose-amended medium (Supplementary Fig. 3). Expression of *gfp* from the xylose promoter was shown to be lowered when both glucose and xylose sugars are present, likely due to catabolite repression, as observed for other strains of *B. megaterium*^34,35^ (Supplementary Fig. 4). Based on the rapidity, strength, and uniformity of induction, the remaining work presented herein utilized the pXyl promoter. Plasmid retention was assessed, as the literature is mixed regarding long-term exogenous plasmid stability in *B. megaterium*^36,37^. Plasmid retention ( > 87%) was verified after 74 h of culturing by counting colony forming units (CFUs) on media with and without antibiotic selection as well as confirming the presence of plasmid elements for individual colonies (Supplementary Table 2 and Supplementary Fig. 5).

### Heterologous protein production in SR7 grown under scCO_2_

The anaerobic functionality of the xylose promoter was next established for SR7 grown under 0.1 MPa CO_2_ (i.e., atmospheric pressure) as well as a 10 MPa headspace of scCO_2_. Several challenges were anticipated when moving from aerobic to scCO_2_-based culturing, including slower and less predictable growth due to stresses associated with scCO_2_, and reduced maintenance of non-native plasmids through sporulation, germination, and outgrowth. Since the formation of the GFP chromophore is oxygen dependent, the β-galactosidase protein (LacZ) from *E. coli* was selected as a reporter for anaerobic cultures due to its previous use in *B. megaterium*^32^, its oxygen independence, and its easy and sensitive detection via enzymatic assay.

**Fig. 3:**
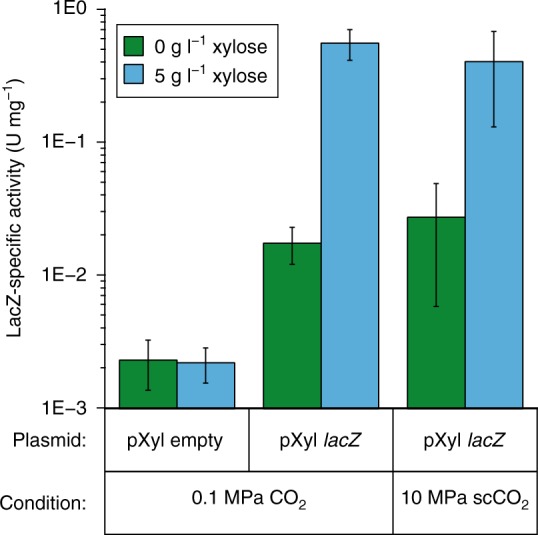
β-Galactosidase activity for SR7 cultures grown under 0.1 MPa CO_2_ and 10 MPa scCO_2_ at 37 °C. Specific β-galactosidase activity measured in lysates of SR7 cultures that contain an empty pXyl plasmid or pXyl *lacZ*. Activity was normalized to total protein concentration in the lysate to control for growth differences and differential cell lysis. Errors represent standard deviation of biological replicate cultures (*n* > 4). For cultures grown under 0.1 MPa CO_2_, overnight anaerobic cultures grown from spores were subcultured into CO_2_-evacuated vials to a uniform optical density. Induction with xylose occurred 2 h after subculture, and the time point for LacZ activity measurement was taken 4 h post induction. For cultures grown under 10 MPa scCO_2_, SR7 pXyl *lacZ* spores were loaded into stainless steel culture vessels with and without xylose inducer. Cultures were grown for 21 days and microscopy was used to determine which samples showed at least a tenfold increase in cell number prior to measuring LacZ activity. Source data are provided as a Source Data file

After confirming heterologous *lacZ* expression (Fig. 3) and plasmid maintenance under 0.1 MPa CO_2_ (Supplementary Note 2), the functionality of the pXyl promoter was assessed for SR7 pXyl *lacZ* cells grown under 10 MPa scCO_2_ at 37 °C in semi-defined medium containing glucose as a carbon source and l-alanine as a germination inducer^25^. Xylose was added as an inducer at the time of inoculation rather than during early exponential growth since high-pressure cultivation of SR7 in stainless steel columns precludes non-destructive amending of samples during cultivation. After 21 days of incubation under scCO_2_, LacZ activity was measured in crude lysates derived from columns that displayed growth. Cells grown under 0.1 MPa and 10 MPa CO_2_ with xylose displayed similar LacZ specific activities of 0.56 and 0.40 U mg^–1^, respectively (Fig. 3). The 15-fold increase in LacZ expression under 10 MPa relative to uninduced cultures (*p* = 0.0059) is similar in magnitude to the increase in induced-expression observed under 0.1 MPa (Fig. 3).

Growth of SR7 from endospores under scCO_2_ has been defined as at least a tenfold increase in cell number and presence of vegetative cells, as determined by direct cell counts using fluorescence microscopy^25^. SR7 pXyl *lacZ* cultures amended with xylose grew in 8 of 55 columns (15%) while growth was observed in 5 of 34 unamended columns (15%) (Supplementary Table 3), which together represent a 4.4-fold decrease in growth frequency relative to wild-type SR7 (64%) under the same conditions (i.e., 5 ml culture volume with 3 × 10^5^ spores ml^–1^ inoculum and 5 ml scCO_2_ headspace)^25^. Previous studies have shown bacteria transformed with exogenous plasmids are more susceptible to cell death than wild-type strains, especially under harsh culture conditions^38,39^, consistent with our results. Improving growth of genetically modified SR7 remains an important target for further optimization.

### Alcohol production and optimization in aerobically grown SR7

**Fig. 4:**
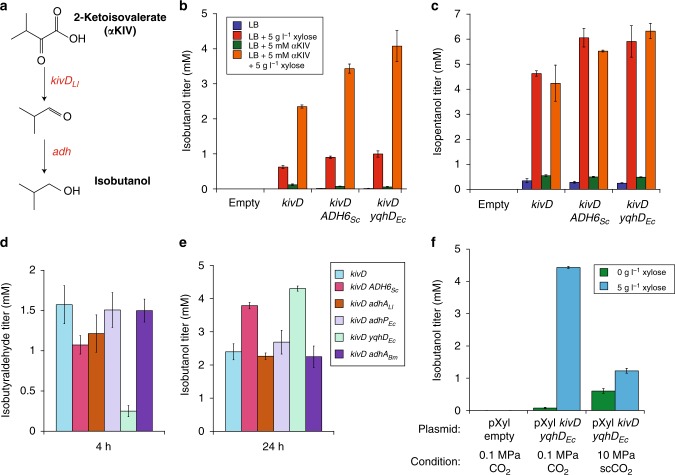
Isobutanol and isopentanol production in SR7. **a** Two-step enzyme pathway to convert the valine intermediate 2-ketoisovalerate (αKIV) to isobutanol using the decarboxylase enzyme KivD from *L. lactis* and an alcohol dehydrogenase. **b** Evaluation of isobutanol production at 24 h post induction in SR7 with an empty pXyl plasmid, pXyl *kivD*, pXyl *kivD ADH6*_*Sc*_, or pXyl *kivD yqhD*_*Ec*_, where ADH6 and YqhD are alcohol dehydrogenases from *S. cerevisiae* and *E. coli*, respectively. Cultures were grown aerobically in LB medium in the presence and absence of 5 g l^–1^ xylose as an inducer and 5 mM αKIV as substrate for the pathway. For all aerobic experiments, error bars represent the standard deviation of biological triplicate cultures. **c** Co-production of isopentanol measured in SR7 samples shown in **b**. **d** Accumulation of the isobutyraldehyde intermediate at short culture times, 4 h post induction. Alcohol dehydrogenases ADH6 from *S. cerevisiae*, AdhA from *L. lactis*, AdhP from *E. coli*, YqhD from *E. coli*, and AdhA from *B. megaterium* SR7 were evaluated to decrease the buildup of isobutyraldehyde intermediate for aerobic cultures fed 5 mM αKIV and induced with xylose. **e** Production of isobutanol at 24 h post induction for the cultures shown in **d**. **f** Production of isobutanol for SR7 cultures grown under 0.1 MPa CO_2_ (biological triplicate cultures) and 10 MPa scCO_2_ at 37 °C. Under scCO_2_, cultures with at least a tenfold increase in cell number, as enumerated by microscopy, were analyzed for alcohol production, sugar consumption and fermentation product generation. Average isobutanol titers from the aqueous phase of cultures showing at least 1 mM glucose consumption (classified as high activity; Supplementary Fig. 11) are provided. Source data are provided as a Source Data file

An isobutanol bioproduction pathway was developed in SR7 due to previous success at achieving high yields and titers of this compound in *B. subtilis*^31,40^, and the fact that the isomer *n*-butanol can be effectively extracted from a biphasic aqueous-scCO_2_ reactor^12^. Furthermore, isobutanol generation from 2-ketoisovelerate (αKIV) is expected to require heterologous expression of only one enzyme, α-ketoisovalerate decarboxylase (KivD from *L. lactis*)^27^ (Fig. 4a), since the SR7 genome encodes eight predicted alcohol dehydrogenases^25^. For SR7 grown in LB medium under aerobic conditions, isobutanol production was only observed for cells that contained *kivD*, with approximately 50% yield on αKIV in cultures that were both induced with xylose and fed the αKIV substrate (Fig. 4b). To increase product yield, the alcohol dehydrogenase ADH6_*Sc*_ from *S. cerevisisae* was overexpressed in tandem with KivD, resulting in approximately 70% yield of isobutanol on αKIV for xylose-induced cells (Fig. 4b). In addition to the target isobutanol molecule, isopentanol (Fig. 4c and Supplementary Figs. 6 and 7) and phenylethyl alcohol (PEA) (Supplementary Fig. 8a) production were also observed, with the isopentanol titer being greater than that of isobutanol. Accumulation of these products is not surprising given the known promiscuity of KivD towards other amino acid synthesis pathway intermediates^27^ (Supplementary Fig. 8b).

Biosynthesis of alcohols entails conversion of aldehyde intermediates (Fig. 4a) that tend to be highly soluble in scCO_2_, partitioning approximately tenfold more readily into scCO_2_ than their cognate alcohols^7^. Thus, any accumulation of aldehyde is hypothesized to result in premature extraction of this compound under scCO_2_, resulting in lower alcohol production. Under aerobic conditions at short induction times of 4 h, isobutyraldehyde buildup was observed, even for cells that contained the alcohol dehydrogenase *ADH6*_*Sc*_ (Fig. 4d). Four additional alcohol dehydrogenases were screened for reduced aldehyde accumulation: three previously shown to be functional within the context of the isobutanol pathway^41^, and a predicted alcohol dehydrogenase native to SR7 (AdhA_*Bm*_) with high homology to AdhA_*Ll*_ from *L. lactis*. The YqhD enzyme from *E. coli*, known to be highly effective for producing isobutanol^41,42^, was identified as the superior variant, with the lowest amount of aldehyde accumulation at short culture times (4 h) (Fig. 4d), over 80% yield of isobutanol from αKIV (Fig. 4e), and maintained capacity for isopentanol production **(**Fig. 4c).

The optimized two-enzyme isobutanol production pathway was evaluated aerobically in semi-defined medium containing 4 g l^–1^ glucose and 5 g l^–1^ xylose (conditions previously shown to support heterologous enzyme production in SR7 under anaerobic conditions). The final isobutanol titer was approximately half of that observed in LB medium (Supplementary Fig. 9a), possibly due to lower heterologous enzyme expression and more αKIV substrate being directed towards biomass in the lower nutrient semi-defined medium. Increasing the glucose concentration to 10 g l^–1^ in the semi-defined medium increased the titers of all three alcohols, suggesting that cellular resources may be directed towards these products when carbon is provided in greater excess (Supplementary Fig. 9b). When neither glucose nor xylose were supplied, minimal biomass (OD_600_ < 0.1) or product accumulated (Supplementary Fig. 9c), indicating that neither growth nor alcohol production is due to trace amounts of yeast extract (50 mg l^–1^). Xylose alone enabled high cell density growth (OD_600_ > 6.5), and in the case of isopentanol, almost 1 mM production, with a further increase in titer upon adding αKIV substrate (Supplementary Fig. 9c). When combined with xylose, the addition of 100 mM alanine (used under anaerobic CO_2_ conditions for endospore germination) increased cell growth (OD_600_ > 15) and more than doubled isobutanol production (Supplementary Fig. 9d), likely due to the deamination of alanine to pyruvate, a precursor for isobutanol via the valine-synthesis pathway and part of central carbon metabolism.

### Isobutanol production in SR7 under scCO_2_

After optimizing aldehyde conversion and alcohol production for SR7 under aerobic conditions, functionality of the two-step isobutanol pathway was assessed in semi-defined medium under anaerobic conditions (Fig. 4f). Seed cultures of SR7 pXyl *kivD yqhD*_*Ec*_ cells passaged under 0.1 MPa CO_2_ into fresh medium amended with xylose and 5 mM αKIV substrate generated 4.43 mM isobutanol after 48 h of incubation. The final isobutanol titer under anaerobic 0.1 MPa CO_2_ headspace is greater than the 2 mM isobutanol generated by aerobic cells grown in the same medium conditions. Isopentanol and PEA titers were 1.8 and 0.21 mM, respectively (Supplementary Fig. 10). As expected, no isobutanol or isopentanol was observed for SR7 cultures that contained an empty plasmid (Fig. 4f and Supplementary Fig. 10).

Biofuel production was next evaluated for SR7 cultures grown under supercritical CO_2_ at 10 MPa and 37 °C. Cultures were grown for 21 days in semi-defined medium from SR7 endospores containing the desired biofuel pathway or empty plasmid, with 4 g l^–1^ glucose, 5 g l^–1^ xylose, 5 mM αKIV, and 100 mM alanine added at the time of inoculation. As was found for SR7 pXyl *lacZ*, the addition of a plasmid resulted in lower frequency growth (27%) as compared to SR7 wild-type (64%)^25^ (Supplementary Table 3). For cultures that displayed growth, glucose consumption was not correlated with increases in cell number (Supplementary Fig. 11a), possibly due to consumption of yeast extract and l-alanine. Glucose consumption was found to correlate strongly with the production of fermentation products lactate and acetate (*R*^2^ = 0.97) (Supplementary Fig 11b), which have previously been shown to accumulate in SR7 cultures grown under scCO_2_^25^. This finding led to classifying samples that consumed more than 1 mM glucose as having high activity, as all of these samples also produced greater than 1 mM combined lactate and acetate (Supplementary Fig. 11b). Other samples that showed at least tenfold growth, but less than 1 mM glucose consumption were classified as having low activity since they neither consumed sugar substrate nor generated meaningful amounts of fermentation products (Supplementary Fig. 11b).

Isobutanol production was measured from the aqueous phase of scCO_2_ cultures grown in the presence and absence of xylose (Supplementary Fig. 12). Xylose-containing high activity cultures produced an average of 1.23 mM isobutanol, whereas those without xylose generated approximately half that amount (Fig. 4f and Supplementary Table 4). Isobutanol titers normalized to cell density were comparable under 0.1 MPa CO_2_ and 10 MPa scCO_2_, at 2.2 × 10^–8^ μmol cell^–1^ and 3.1 × 10^–8^ μmol cell^–1^, respectively (Supplementary Table 5). While the high activity, empty vector control cultures consumed up to 1.9 mM αKIV under 10 MPa scCO_2_, isobutanol was not detected in any of these samples (Supplementary Table 4), an identical outcome to aerobic and 0.1 MPa CO_2_ cultures. The isobutanol yield from αKIV ranged from 37 to 45% in the aqueous phase for xylose-induced high activity biofuel cultures, 2.8-fold higher than for those that lacked xylose (Supplementary Table 4). Isopentanol generation was also observed for samples grown under 10 MPa scCO_2_, reaching an average titer of 0.31 mM (Supplementary Table 4), while only trace amounts of PEA were detected under scCO_2_. The amount of isobutanol produced by low activity cultures was on average 35-fold less than for the high activity cultures (Supplementary Table 4).

### Extraction of isobutanol using scCO_2_

Inspired by our demonstration of microbially generated alcohols under scCO_2_, we attempted to recover product from SR7 cultures using scCO_2_. Partitioning of biologically produced isobutanol into the scCO_2_ headspace was observed for the aforementioned SR7 cultures (Supplementary Fig. 13), albeit at marginal concentrations. Isopentanol was not detected in the headspace samples. Our proof of concept demonstration for isobutanol extraction motivates adoption of two engineering solutions to increase recovery: (1) use of flowing scCO_2_ to increase mass transfer and (2) controlled de-pressurization to capture scCO_2_-solubilized alcohol. To evaluate these solutions, we assessed the recovery of isobutanol in the absence of microbial cells using a stirred two-phase reactor that is designed for in situ extraction using scCO_2_ stripping (Supplementary Fig. 14), as previously done for *n-*butanol and *n*-pentanol^12^. Similar to straight-chained alcohols, the rate of isobutanol extraction from aqueous solutions increases with low scCO_2_ flow rates, reaching a maximum at a flow rate of 3.2 ml min^–1^, most likely due to a tradeoff between convective and diffusive mass transfer. Isobutanol extraction rate and efficiency using scCO_2_ was found to be approximately 25% greater than that for *n*-butanol (Supplementary Fig. 14). Taken together, these data indicate that scCO_2_ is effective for rapid extraction of isobutanol from dilute aqueous solutions and identifies scale up of cultures to a similarly designed system as a priority for further development.

### Process analysis for extraction using scCO_2_

The experimental component of this study established growth and heterologous bioproduction in the presence of scCO_2_ and extraction of isobutanol into the scCO_2_ phase in a stirred reactor. Next, we constructed a fermentation-extraction process model to assess technological potential using detailed mass and energy balances. The objective of the analysis was calculation of isobutanol recovery energy requirements for comparison with previously reported analyses of butanol recovery using other methods^28^. In practice, Oudshoorn suggested a benchmark of 5 MJ kg^–1^ as the maximum energy requirement for butanol recovery^28^. Unfortunately, previous studies of butanol recovery from dilute fermentation broths ( < 20 g l^–1^) using a wide range of conventional technologies, such as gas stripping, adsorption, and liquid–liquid extraction, report energy requirements in the range of 10–20 MJ kg^–1^^28^. By comparison, Tompsett et al.^12^ estimated the energy demand for the CO_2_ compression required for scCO_2_ extraction of dilute *n*-butanol to be 4.0 MJ kg^–1^. Since CO_2_ compression is expected to represent the majority of the energy required by the scCO_2_ extraction process, these results^12^ suggest that the energy requirement of scCO_2_ extraction might compare favorably with alternative technologies — especially since efforts to minimize CO_2_ compression requirements were not considered. Moreover, this previous study^12^ did not analyze the performance of an integrated fermentation-extraction process since bioproduction in the presence of scCO_2_ has never been reported prior to this work. Accordingly, the current study evaluated approaches to reduce CO_2_ compression requirements, the advantages associated with coupled fermentation-extraction (Supplementary Note 3), and the intrinsic performance of the scCO_2_ extraction process.

**Fig. 5:**
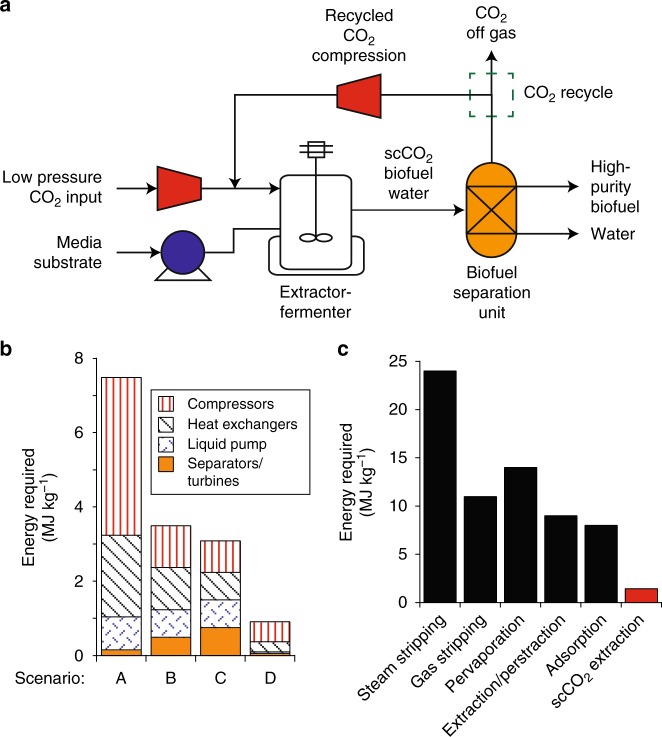
Isobutanol energy recovery requirements for integrated fermentation-extraction under scCO_2_. **a** Simplified process schematic of the envisioned scCO_2_ bioprocess used to develop an Aspen Plus model, including compression of CO_2_ to scCO_2_, fermentation/extraction, de-pressurization/biofuel recovery, and CO_2_ recycle. Further detail is provided in Supplementary Fig. 15a. **b** Energy requirement for isobutanol recovery as MJ kg^−1^ for several scenarios. Separate fermentation and scCO_2_ recovery processes represent the base case (scenario A). Scenario B is coupled fermentation and extraction without use of partial extraction stream de-pressurization or scCO_2_ recycle. Scenario C de-pressurizes the extraction stream to 6.5 MPa to induce formation of an isobutanol-rich stream, which permits recycling of compressed CO_2_, set at 50% (by mass). Scenario D again de-pressurizes to 6.5 MPa and recycles the maximum possible amount of CO_2_ (87%) that results in no accumulation of CO_2_ in the system. Values for **b** can be found in Supplementary Table 6. **c** Comparison of the energy requirements for isobutanol production found for the scCO_2_ process relative to published literature values for alternative in situ recovery methods^28^. The scCO_2_ extraction energy requirement in **c** is for coupled fermentation and extraction with partial de-pressurization and full recycle (i.e., Scenario D in **b**). Source data are provided as a Source Data file

With emphasis on evaluating energy demand for recovering a high-purity product, we developed an Aspen Plus simulation model (Fig. 5a and Supplementary Fig. 15a) to analyze a process consisting of integrated fermentation and extraction using scCO_2_. Isobutanol extraction performance was simulated using data reported in the current work, literature data on *n*-butanol extraction^12,43,44^, and established thermodynamic properties^45,46^ (Supplementary Note 3). Sensitivity analysis revealed that the most influential parameter in determining isobutanol energy requirement was the CO_2_:isobutanol feed ratio since it directly relates to the amount of CO_2_ that must be compressed for extraction (Supplementary Fig. 15b and Supplementary Note 3). Combining our isobutanol extraction data with values reported on *n-*butanol^44^, we calculate that 99.73% isobutanol recovery could be achieved at a CO_2_:isobutanol feed ratio of 3:1 as a base case. To reduce CO_2_ compression energy demand, product recovery via partial de-pressurization of the CO_2_ stream was explored, finding > 94% pure isobutanol product could be recovered by partially de-pressurizing the stream exiting the fermenter to between 3.5 and 6.5 MPa^45^. In this pressure range, liquid–liquid phase splitting occurs, resulting in isobutanol-rich and water-rich liquids^45^. Partial de-pressurization allows recycling of a high-pressure CO_2_ stream, further reducing CO_2_ compression demand and thereby energy consumption (Supplementary Fig. 15b). Lastly, the sensitivity of the process to the ratio of recycled to purged CO_2_ was investigated using an optimized intermediate pressure of 6.5 MPa (Supplementary Fig. 15b). Increasing the CO_2_ recycle amount decreased energy requirements nearly linearly, with the best performance observed at the limit of 87% recycle (Supplementary Fig. 15c).

Based on these sensitivity analyses, four scenarios were assessed using the process model, with specific emphasis on evaluating the extent to which partial de-pressurization and CO_2_ recycling could be used to decrease energy requirements of isobutanol extraction (Fig. 5b and Supplementary Table 6). Atmospheric-pressure fermentation followed by scCO_2_ extraction (scenario A) required an estimated 6.80 MJ kg^–1^, with compression accounting for approximately 66% of the energy demand. An integrated process, with complete de-pressurization to 0.1 MPa and no CO_2_ recycling (scenario B), reduced the energy burden to 4.4 MJ kg^–1^, with most of the improvement stemming from reduced compression requirements. Intermediate de-pressurization to 6.5 MPa combined with 50% CO_2_ recycle (scenario C)^43^, further reduces the energy requirement to 2.4 MJ kg^–1^, again largely due to diminished compression requirements. Finally, placing the CO_2_ recycle ratio at its maximum value (87%) (scenario D) and de-pressurizing the extraction stream to 6.5 MPa reduces the energy requirement to 1.4 MJ kg^–1^. As expected from previous arguments, the newly established energy requirement is lower than that previously reported for stand-alone fermentation and scCO_2_ extraction, and much less than any figure reported for conventional technologies such as gas stripping, liquid–liquid extraction, or adsorption (Fig. 5c). Although the energy requirement estimated here for integrated extraction-fermentation has not yet been demonstrated in practice, the current finding motivates further evaluation, with our model serving as a basis for follow-on techno-economic, emissions, and life cycle assessment studies.

## Discussion

In this work, we report the production of alcohol biofuels under supercritical carbon dioxide. Using an environmentally isolated strain (*Bacillus megaterium* SR7) native to a deep subsurface CO_2_ reservoir, a transformation protocol was developed and heterologous protein expressed from three promoters. Furthermore, inducible β-galactosidase production was observed for cells grown under scCO_2_. SR7 was engineered to produce isobutanol from αKIV through a two-step metabolic pathway that simultaneously produced isopentanol. Both isobutanol and isopentanol have potential as drop-in biofuels or fuel additives, with comparable energy densities (29 MJ l^–1^ and 28 MJ l^–1^, respectively) and research octane numbers (98 and 105)^47^. Similar to isobutanol, isopentanol is predicted to be highly miscible in scCO_2_^12^. For cultures grown under scCO_2_ that showed high activity, isobutanol titers were greater than 1 mM, with 42% average yield on consumed αKIV substrate. Importantly, while total titers were reduced at 10 MPa, specific titers under scCO_2_ were similar to those observed under 0.1 MPa conditions, at 2.2 × 10^−8^ μmol cell^–1^ and 3.1 × 10^−8^ μmol cell^–1^, respectively. Recovery of microbially generated isobutanol using scCO_2_ was demonstrated, but limited, due to both the configuration of the high-pressure culturing vessels and the low titers achieved. Separation of dilute isobutanol from aqueous media using scCO_2_ is possible using a two-phase extractor-fermenter, as previously observed for straight-chain alcohols^12^. Lastly, process analysis shows that using scCO_2_ to extract biofuels is energetically feasible and comparable, if not better, than other in situ extraction techniques if sufficiently high product titers can be achieved. As such, this work serves as a starting point for bioproduction under scCO_2_ with numerous avenues for continued optimization.

Further development of the genetic toolbox for SR7 will enable future metabolic engineering of SR7 for enhanced biochemical production. Isobutanol pathway optimization was briefly explored by screening five alcohol dehydrogenases and selecting the variant that resulted in highest substrate conversion ( > 80%) and minimal aldehyde accumulation at short incubation times. Additionally, isopentanol and phenylethyl alcohol production were observed through the simple addition of a decarboxylase, possibly due to upregulation of the leucine and phenylalanine synthesis pathways in SR7. Other strains of *B. megaterium* are known to accumulate the amino acids lysine^48^ and possibly valine^49^; however, evidence for leucine and phenylalanine accumulation was not previously observed^49^. The leucine biosynthesis pathway may become a starting point for future engineering efforts to increase isopentanol titers or make additional bioproducts due to the already presumably high flux of the pathway^50^. To ensure that coupled fermentation-extraction technology is utilized to its full potential, initial screening of subsequent compounds to be made by SR7 will be determined based on their preferential partitioning into scCO_2_ and toxicity to bioproduction hosts.

Taking advantage of scCO_2_ for properties beyond its well-established extractive^12^ and sterilizing capabilities^13^ would confer additional utility to this technology. For example, scCO_2_ has been used for the depolymerization of lignocellulosic biomass to release fermentable sugars^51^. Combining the two technologies could provide an inexpensive substrate for SR7 growth and bioproduction. Carboxylation reactions may also be possible in SR7, directly using CO_2_ or HCO_3_^–^ found in solution for bioproduction^52,53^. Even with the low solubility of scCO_2_ in water^54^, the approximate HCO_3_^–^ concentration of 1.1–1.3 mM (based on Henry’s law and the reaction of CO_2(aq)_ with water) is advantageous relative to other studies that use air as a CO_2_ source for carbon fixation through heterologous overexpression of a carboxylase^55,56^. SR7 contains several predicted carboxylases, including pyruvate carboxylase, two PEP carboxylases and a biotin-dependent acetyl-CoA carboxylase, each of which could serve as pathways to route HCO_3_^–^ into central carbon metabolism^25^. Additionally, the SR7 genome encodes two predicted carbonic anhydrases for converting CO_2(aq)_ to HCO_3_^–^. Lastly, *Bacillus megaterium* strains are known for their ability to secrete proteins^57^, conferring further biotechnological utility to this host for ex vivo reactions.

One of the limitations to using scCO_2_ for bioprocessing is the slow and infrequent growth observed for SR7, especially when bearing an exogenous plasmid. Significant improvements were previously made for scCO_2_ culturing using SR7 through media optimization and recognition of endospore germination as a key step for acclimation of this strain to conditions associated with scCO_2_ exposure^25^. Future work will be conducted to better understand the factors limiting growth of SR7 under scCO_2_ and to select for strains of SR7 that demonstrate more robust and rapid outgrowth into vegetative cells. Enhanced understanding and substantial improvement of SR7 growth will similarly inform economic, environmental, and energy analysis of the integrated process, due to the inter-relationships between growth, productivity, titer, scCO_2_ volumes and extraction efficiency, energy requirements, and ultimately cost. Numerous studies have shown the ability to improve the tolerance of microbes to harsh environments, including through the use of adaptive laboratory evolution^58^. Many of these studies, however, found that increased tolerance of strains does not necessarily correlate with enhanced specific titers when used for bioproduction^59–61^. For SR7, the fact that specific titer is comparable under atmospheric and scCO_2_ conditions suggests that scCO_2_ does not impair specific productivity and that improvements in titer observed under 1 atm CO_2_ will positively translate into higher titers under scCO_2_. Using the plasmid bearing strains in this work, future selection of SR7 mutants that have a higher growth capacity despite the burden associated with plasmid maintenance and expression may also be possible^62^. Additionally, genomic integration could alleviate plasmid-maintenance burden for SR7 strains, and recently developed Cas9-based genomic modification strategies for *B. subtilis* strains^63,64^ provide a starting point for integrating and removing desired and competing pathways, respectively, in SR7.

The isolation, characterization and genetic engineering of an scCO_2_-tolerant strain of *B. megaterium* establishes exciting biotechnological opportunities for in vivo high-pressure CO_2_ bioprocessing coupled with in situ product extraction. While low-frequency growth from *Bacillus* spp. spores^22^ and enzymatic catalysis^15^ have previously been shown under scCO_2_, this work demonstrates combined heterologous enzyme expression and bioproduct synthesis under these harsh conditions. Proven scCO_2_ stripping of biofuels and favorable energy requirements of scCO_2_-based bioprocessing lend credibility to the adoption of this process for commercial applications, motivating continued work to improve titers, yields, and productivities. Achieving such improvements would enable demonstration of the integrated extraction-fermentation process at a larger scale, facilitating collection of data needed to form the basis for full techno-economic and life cycle analyses, and more detailed comparisons to other extraction strategies and renewable energy sources.

## Methods

### Plasmid construction

For plasmid construction (Supplementary Table 7), restriction enzymes, T4 DNA ligase, and Q5 High-Fidelity PCR MasterMix were purchased from NEB and used according to manufacturer recommendations. The pXyl *gfp* plasmid is based on the pRBBm34 shuttle vector^65^ (purchased from Addgene), which contains a xylose-inducible promoter, the xylose repressor (*xylR*) under control of its native *B. megaterium* promoter, ampicillin resistance marker (Amp^r^) for selection in *E. coli*, tetracycline resistance marker (Tet^r^) for selection in *B. megaterium*, and two origins of replication. The gene encoding SuperFolder GFP was PCR amplified from the pTRC99a *gfp*_*SF*_ (lab stock) template using the oligonucleotides *Spe*I *gfp* for and *gfp Sph*I rev (Supplementary Table 8). The resulting PCR product and pRBBm34 plasmid were digested with *Spe*I and *Sph*I, and resulting fragments ligated to create pXyl *gfp*. The pXyl empty plasmid was generated by adding the DNA sequence 5ʹ-atttaatgttgatgaaagctggtctggtgtcaaaaataatga-3ʹ between the *Spe*I and *Sph*I restriction enzyme sites. Ligated plasmid products were used to transform DH5α and plated on LB agar containing 100 μg ml^–1^ carbenicillin (Sigma). Plasmids were purified (Zippy plasmid miniprep kit, Zymo research) prior to verification by DNA sequencing (Genewiz) using appropriate sequencing oligonucleotide primers (Supplementary Table 8).

The xylose repressor and promoter of pRBBm34 were replaced with a hyperspank promoter and *lacI* using circular polymerase extension cloning (CPEC). The pRBBm34 plasmid was PCR linearized with two sets of primers to remove *xylR* and the xylose promoter: pRBBm34 for/*bla* rev and *bla* for/pRBBm34 rev. The hyperspank promoter and *lacI* were PCR amplified from pDR111^66^ (gift from Alan Grossman, MIT Department of Biology) using primers pRBBm34-pHysp for and *lacI*-pRBBm34. A standard CPEC protocol^67^ was used to assemble the three PCR products into the pHysp empty plasmid. *Gfp* was PCR amplified from pXyl *gfp* using primers *Sal*I RBS *gfp* for and *gfp Nhe*I rev. The *gfp* PCR product and pHysp empty were digested with *Sal*I and *Nhe*I, and the fragments were assembled to make pHysp *gfp*. To generate the p43 empty and p43 *gfp* plasmids, the pXyl empty and pXyl *gfp* plasmid (respectively) were PCR linearized with oligonucleotide sets RBS-start for/*bla* rev and *bla* for/pRBBm34 rev. The p43 promoter was PCR amplified from *Bacillus subtilis* strain KS438 using primers pRBBm34-p43 for and p43-RBS-start rev. The two p43 plasmids were created by assembling the three PCR fragments using CPEC.

All the remaining plasmids were made using standard restriction enzyme digest and ligation construction; the pairs of restriction enzymes used are indicated in the oligonucleotide primer names. The gene encoding β-galactosidase from *E. coli* was PCR amplified from the plasmid pKVS45 *lacZ*_LVA (lab stock) using primers *Spe*I *lacZ*_*Ec*_ for and *lacZ*_*Ec*_*Sph*I rev. The ketoisovalerate decarboxylase gene from *L. lactis* was amplified from pCOLA (Fjoh-2967) (*kivD*)^68^ with primers *Spe*I *kivD*_*Ll*_ for and *kivD*_*Ll*_*Bam*HI *Sph*I rev for the construction of pXyl *kivD*_*Ll*_. The *ADH6* gene from *Saccromyces cerevisiae* was amplified from pACYC (*car sfp*) (*ADH6*_*Sc*_)^68^ using primers *Bam*HI RBS *ADH6*_*Sc*_ for and *ADH6*_*Sc*_*Sph*I rev to make the plasmid pXyl *kivD*_*Ll*_*ADH6*_*Sc*_. For all the alcohol dehydrogenases studied, the RBS 5ʹ-agggggaaa-3ʹ was used. The *adhA*_*Ll*_ gene was amplified from the *L. lactis* subsp. *lactis* (ATCC 19435D) genome (primers: *Bam*HI *adhA*_*Ll*_ for and *adhA*_*Ll*_*Sph*I rev), *adhP*_*Ec*_ and *YqhD*_*Ec*_ from the *E. coli* MG1655 genome (primers: *Bam*HI *adhP*_*Ec*_ for and *adhP*_*Ec*_*Sph*I rev; *Bam*HI *yqhD*_*Ec*_ for and *yqhD*_*Ec*_*Sph*I rev, respectively), and *adhA*_*Bm*_ from the *B. megaterium* SR7 geneome (primers: *Bam*HI *adhA*_*Bm*_ for and *adhA*_*Bm*_*Sph*I rev). All alcohol dehydrogenases were ligated between the *Bam*HI and *Sph*I sites in pXyl *kivD*_*Ll*_.

### Transformation of SR7 and verification

Protoplast transformation of SR7 was adapted from an established method by Biedendieck et al.^29^. All buffers described in this protocol were identical to those in Biedendieck et al.^29^ and were made with chemicals from Millipore-Sigma unless otherwise described. An overnight culture of SR7 was grown from the inoculation of 50 ml of LB medium (BD Difico) with a single SR7 colony, and was incubated at 37 °C and 250 r.p.m. SR7 was subcultured at a dilution of 1:100 in fresh LB medium and grown at 37 °C and 250 r.p.m. until an OD_600_ reading of 1.0 was reached (~3 h) (Implen NanoPhotometer). Cells were collected by centrifugation at 4000 × *g* for 10 min and the cell pellet was retained. The cell pellet was resuspended in 5 ml 1x SMMP buffer (2.32 g l^-1^ L-malic acid (Alfa Aesar), 1.6 g l^-1^ NaOH, 4.06 g l^-1^ MgCl_2_•6H_2_0, 171.16 g l^-1^ sucrose, 17.5 g l^-1^ antibiotic medium number 3 (AB3) (BD Difico)). Lysozyme (Sigma) was added to a concentration of 30 μg ml^–1^, which was tenfold lower than used in previous methods^29^. Protoplast formation was performed at 37 °C for 10 min at 70 r.p.m. and was visualized by microscopy.

Protoplasts were collected by centrifugation at 1300 r.p.m. for 10 min, followed by a wash with 5 ml 1x SMMP. Protoplasts were resuspended gently in 5 ml 1x SMMP and were aliquoted into 0.5 ml fractions. Each sample was transformed with 3 μg freshly prepared plasmid DNA (Zippy plasmid miniprep kit, Zymo research) and 1.5 ml of a 20% PEG 8000 (Promega) solution in 1x SMM (2.32 g l^-1^ L-malic acid (Alfa Aesar), 1.6 g l^-1^ NaOH, 4.06 g l^-1^ MgCl_2_•6H_2_0, 171.16 g l^-1^ sucrose). Samples were incubated with gentle mixing for 2 min, followed by addition of 5 ml 1x SMMP and centrifugation to collect the transformed protoplasts. The protoplasts were resuspended in 0.5 ml 1x SMMP and incubated at 37 °C for 1.5 h at 100 r.p.m. to recover. Transformed protoplasts were plated in a soft agar overlay^29^ on dried and pre-warmed LB agar plates containing 5 μg ml^–1^ tetracycline (Calibiochem). Additionally, protoplasts were plated in agar overlays on LB agar without antibiotic to determine viability. Plates were incubated for two days at 30 °C.

Confirmation of SR7 transformation was carried out using several techniques including colony PCR, streaking colonies on fresh LB agar plates containing 5 μg ml^–1^ tetracycline, sequencing isolated plasmid DNA, and back transforming plasmid DNA into *E. coli*. For colony PCR, colonies were picked and boiled for 10 min to serve as template for the PCR reaction using OneTaq Quick-Load Master Mix (NEB) and appropriate sequencing primers (Supplementary Table 8). Plasmid DNA was isolated from SR7 using a standard plasmid isolation protocol and materials (Zippy plasmid miniprep kit, Zymo research), except 10 μg ml^–1^ lysozyme was added for the initial resuspension step with a 1 h incubation at 37 °C to promote cell lysis. Standard Sanger sequencing of isolated plasmids was used to verify identity (Genewiz). Purified plasmids were also backtransformed into *E. coli* DH5α, which were subsequently plated on LB agar containing 100 μg ml^–1^ carbenicillin. Plasmid stability was assessed by isolating the backtransformed plasmid DNA and restriction enzyme digestion with *Eco*RI (NEB). For a positive control, purified pXyl *gfp* directly from *E. coli* DH5α was also digested with *Eco*RI. Lastly, SR7 species and strain identity was confirmed through preparation of genomic DNA (Wizard genomic DNA purification kit, Promega) and sequencing of the PCR amplified 16s rRNA and *xylE* genes, respectively. The 16s fragment was PCR amplified using Q5 High-Fidelity MasterMix (NEB) and the 27F and 1492R primers (Supplementary Table 8); the *xylE* gene was amplified using *xylE*_*Bm*_ seq for and *xylE*_*Bm*_ seq rev primers.

### Aerobic SR7 culturing

SR7 was cultured aerobically in complex LB medium and a semi-defined M9-based medium. The semi-defined medium consisted of 1x M9 salts (Sigma) (7 g l^–1^ Na_2_HPO_4_•7H_2_O, 3 g l^–1^ KH_2_PO_4_, 0.5 g l^–1^ NaCl, 1 g l^–1^ NH_4_Cl), 2 mM MgSO_4_, 0.04 mM MgCl_2_, 0.05 g l^–1^ yeast extract (BD Difico), 1x trace metals supplement^69^ (5 mg l^–1^ Na_2_(EDTA), 0.2 mg l^–1^ NiSO_4_•6H_2_O, 0.5 mg l^–1^ CoCl_2_•6H_2_O, 0.1 mg l^–1^ H_2_FeO_3_, 1 mg l^–1^ FeSO_4_•7H_2_O, 0.1 mg l^–1^ H_3_BO_3_, 1 mg l^–1^ ZnCl_2_, 0.1 mg l^–1^ NaMoO_4_•2H_2_O, 0.4 mg l^–1^ AlCl_3_•6H_2_O, 1 mg l^–1^ MnCl_2_•4H_2_O, 0.3 mg l^–1^ Na_2_NO_4_•2H_2_O, 0.2 mg l^–1^ CaCl_2_), and desired concentration of glucose or xylose. For SR7 strains that contained a plasmid, the medium was amended with 5 μg ml^–1^ tetracycline.

Overnight cultures were started in desired medium by picking colonies from freshly grown LB agar plates with 5 μg ml^–1^ tetracycline; different colonies were selected for experiments performed in biological triplicate. Overnight cultures (5 ml) were incubated for approximately 16 h at 37 °C and 250 r.p.m.. For *gfp* production, SR7 was subcultured into 1 ml of fresh medium using 20 μl of an overnight culture into a 48-well flower-plate (m2p labs). Plates were sealed with porous adhesive film for culture plates (VWR) to reduce evaporation, and cultures were grown in a BioLector (m2p labs) at 1200 r.p.m., 37 °C, and 80% humidity. The BioLector continuously monitored (15 min cycle) OD and GFP fluorescence. OD measurements from the BioLector were converted to OD_600_ measurements with a standard pathlength of 1 cm by generating a standard curve using exponentially grown SR7 cells and comparing a dilution series on the BioLector and Implen spectrophotometer. Protein induction occurred 3 h after subculturing (OD_600_ ~0.4) by adding xylose to a final concentration of 5 g l^–1^ for strains that contain the pXyl plasmid, and IPTG to 1 mM for strains containing the pHysp plasmid.

For alcohol production experiments, identical medium and starter culture procedures were used. Overnight cultures were subcultured to an OD_600_ of 0.05 into 3 ml fresh medium in a 50 ml screw-capped tube (Pyrex) to avoid loss of volatile compounds. Protein induction occurred when the OD_600_ of the culture reached approximately 0.4 by adding xylose to a final concentration of 5 g l^–1^ for strains that contain the pXyl plasmid. At the time of induction, αKIV was added to a final concentration of 5 mM. Alcohol production and isobutyraldehyde intermediate accumulation was measured 24 h and 4 h post induction, respectively. Cultures were centrifuged at 21,000 × *g* for 5 min to obtain cell-free supernatants. Alcohols and isobutyraldehyde were extracted from the cell-free supernatant using a 1:1 ratio of GC-grade ethyl acetate (Sigma), vortexing vigorously for 5 min, centrifuging at 21,000 × *g* for 5 min, and recovering the ethyl acetate fraction.

### Plasmid maintenance

For plasmid maintenance studies, a 25 ml culture of SR7 pXyl *gfp* was grown in LB medium with 5 μg ml^–1^ tetracycline in a 250 ml baffled flask. Cultures were inoculated with 250 μl of an overnight culture and were subsequently incubated at 37 °C and 250 r.p.m. Protein induction occurred at 3 h post subculture using 5 g l^–1^ xylose. For each time point, an OD_600_ reading was recorded, and cells were diluted to a uniform OD_600_ in a black-lined 96-well plate (Costar) using LB medium prior to reading GFP fluorescence on a plate reader (Tecan infinite f200 pro, ex: 485/20 nm, em: 535/25 nm). To confirm plasmid maintenance at 74 h post induction, dilutions of cells were made in 1x M9 salts and were plated on LB agar with and without 5 μg ml^–1^ tetracycline to determine colony forming units. Colony PCR was performed using primers RepU seq for and RepU seq rev to amplify the RepU fragment, and primers pXyl seq for and *gfp* seq rev to amplify *gfp* (Supplementary Table 8). A negative control PCR reaction did not contain DNA template and a positive control used 1 ng μl^–1^ purified pXyl *gfp*.

### FACS and microscopy

Samples for fluorescence activated cell sorting (FACS) were collected 4 h post induction for cells (or 7 h total culturing time for p43-containing cells) grown aerobically in LB medium as described above. Samples were prepared for FACS by diluting 5 μl of culture into 200 μl 1x M9 salts in a 96-well plate. FACS measurements were performed on a FACS Canto (BD) equipped with a high-throughput sampler, and GFP fluorescence was recorded from 488 nm emission laser coupled with the 530/30 nm filter channel. Populations of wild-type SR7 and SR7 with pXyl grown aerobically in LB medium were used to establish forward and side scatter gates to select events corresponding to the morphology of SR7. Fluorescence data was collected for 10,000 cell events for each sample. FACS data was analyzed using FlowJo software and a GFP positive gate was established for fluorescence values above all the negative control samples where SR7 contained an empty plasmid.

Samples for microscopy of SR7 expressing *gfp* were also collected 4 h post induction for cultures grown aerobically in LB medium. Each sample was prepared as a wet-mount on a glass slide below a cover slip that had been prepared with poly-l-lysine (Sigma) to enable cells to adhere. Cells were visualized at 1000x magnification using an epifluorescence microscope (Zeiss Axioscope 2). Cell morphology was observed using phase-contrast microscopy and GFP fluorescence was visualized using a 480/30 nm excitation and 535/40 emission FITC filter set with a 505 nm long pass mirror and an X-Cite Series 200 fluorescence source. Images were captured on a Nikon D100 camera using the NKRemote live-imaging software.

### SR7 endospore preparation

Overnight cultures of SR7 grown in LB medium aerobically were diluted 1:50 in modified G medium (2 g l^–1^ yeast extract, 2 g l^–1^ (NH_4_)_2_SO_4_, 0.025 g l^–1^ CaCl_2_•2H_2_O, 0.5 g l^–1^ K_2_HPO_4_, 0.2 g l^–1^ MgSO_4_•7H_2_O, 0.05 g l^–1^ MnSO_4_•4H_2_O, 0.005 g l^–1^ ZnSO_4_•7H_2_O, 0.005 g l^–1^ CuSO_4_•5H_2_O, 0.0005 g l^–1^ FeSO_4_•7H_2_O, pH 7.1). For SR7 cells that contain a plasmid, both the vegetative cell growth medium and sporulation medium were supplemented with 5 μg ml^–1^ tetracycline. Sporulation was induced by incubating cutures in modified G medium for 72 h at 37 °C. Endospores were collected by centrifugation at 4000 × *g* and 4 °C for 15 min, and sporulation medium was removed by washing 5–10 times with cold wash buffer (0.058 g l^–1^ NaH_2_PO_4_•H_2_O, 0.155 g l^–1^ Na_2_HPO_4_•7H_2_O, 0.01% (vol/vol) Tween 20. Spore preparations were treated at 80 °C for 10 min to kill vegetative cells, and colony forming units were determined pre- and post-heat inactivation. Spore preparations were stored in wash buffer at 4 °C until use^22^.

### SR7 growth under 0.1 MPa CO_2_ and 10 MPa scCO_2_

Prior to anaerobic culturing, SR7 strains were prepared as endospores^22^. For cultures under 0.1 MPa CO_2_ or 10 MPa scCO_2_, a semi-defined M9-based medium consisting of 1x M9 salts, 2 mM MgSO_4_, 0.04 mM MgCl_2_, 0.05 g l^–1^ yeast extract, 0.1x trace metals supplement, and 4 g l^–1^ glucose was degassed with CO_2_ (Air gas) prior to use. Additionally, the medium was supplemented with 0.25 g l^–1^ of reducing agent Na_2_S and 1 mg l^–1^ of the redox indicator resazurin. All anaerobic culture manipulation occurred in an anaerobic chamber (Coy labs) under 0.1 MPa CO_2_.

For low-pressure anaerobic experiments, SR7 cultures were grown under 0.1 MPa CO_2_ headspace in 10 ml of CO_2_-degassed semi-defined medium in 100 ml serum vials with clamped rubber stoppers. Seed cultures were started by inoculating the degassed media in serum vials with 100 μl of previously prepared endospores; for SR7 samples that contain a plasmid, 0.5 μg ml^–1^ tetracycline was added. Cultures were grown for approximately 24 h at 37 °C and 250 r.p.m. Anaerobically grown vegetative cells were passaged into 10 ml of fresh medium to an OD_600_ of 0.05. Cultures were incubated at 37 °C and 250 r.p.m., and protein expression was induced using a final concentration of 5 g l^–1^ xylose 2 h post subculture. For β-galactosidase production experiments, a 1 ml sample was taken 24 h after induction and centrifuged at 21,000 × *g* for 5 min, and cell pellets were retained and stored at –20 °C until analysis. For biofuel generation protocols, 5 mM αKIV was also added at the time of induction. Alcohols were collected 48 h post induction by centrifuging samples at 21,000 × *g* for 5 min and extracting the alcohols using ethyl acetate as was done for the aerobic samples.

For 10 MPa scCO_2_ cultures, M9 semi-defined medium was amended with 100 mM l-alanine (Sigma) to promote germination under pressure^25^. Samples were inoculated with endospores to a final concentration of 3 × 10^5^ spores ml^–1^. Xylose was added to a final concentration of 5 g l^–1^ for cultures to be induced, and αKIV to 5 mM for those to produce biofuels. As described in previous work^22^, high-pressure culturing vessels were constructed of ¾ inch 316 stainless steel tubing for 10 ml total capacity, and fitted with quarter turn plug valves (Swagelok or Hylok). Each column was loaded with 5 ml of medium containing appropriate amendments and endospores, prior to pressurization of the 5 ml remaining headspace to 10 MPa with CO_2_ over a 1 h period. Cultures loaded in steel vessels were grown at 37 °C and shaking at 250 r.p.m. for 21 days prior to de-pressurization. The density of the scCO_2_ phase under these conditions is 0.68 g ml^–1^^70^. The pressure of each column was verified to ensure supercritical conditions were maintained throughout the experiment. 1 ml samples of cells were stained with Syto9 (Invitrogen) and filtered on 0.22 μm polycarbonate filters (Whatman) for cell visualization and enumeration by fluorescence microscopy^25^. The remaining cell culture was centrifuged at 21,000 × *g* for 5 min. For β-galactosidase production experiments, the cell pellets were retained and stored at –20 °C until analysis. For alcohol generation experiments, alcohols were extracted from the cell-free supernatant using ethyl acetate and an identical protocol to what was used for cultures grown under aerobic and 0.1 MPa CO_2_ conditions.

### β-Galactosidase activity assays

Cells from 0.1 MPa CO_2_ and 10 MPa scCO_2_ cultures were lysed by addition of 100 μl of Bacterial Protein Extraction Reagent (B-PER) (Thermo Scientific) to the previously collected cell pellets and vortexing for 30 min at room temperature. Soluble cellular protein was collected by centrifuging the lysed cell pellets for 20 min at 18,500 × *g* and 4 °C. Total protein content for each sample was determined using a Pierce BCA Protein Assay Kit (Thermo Scientific) according to the manufacture’s instructions, and measuring the resulting absorbance at 562 nm (Beckman DU600). To determine protein concentration, samples were compared to a standard curve generated using 0.05–1.0 mg ml^–1^ of bovine serum albumin (BSA) and the same protocol. B-PER negative control samples were also analyzed and subtracted from the total protein content of the lysed samples.

To assay for β-galactosidase activity, lysed culture supernatant was added to assay buffer (0.1 M sodium phosphate buffer pH 8, 10 mM KCl, 1 mM MgSO_4_) to a final volume of 800 μl in a plastic cuvette (VWR). Samples were equilibrated at room temperature prior to the addition of 200 μl of 4 mg ml^–1^ freshly prepared, β-galactosidase substrate o-nitrophenyl-β-D-galactoside (ONPG) (Sigma). Absorbance at 420 nm was collected every 15 s for 10 min using a Beckman DU600 spectrophotometer. The volume of lysed culture was adjusted to ensure the concentration of ONPG was not limiting during the assay. The initial rate of absorbance change was calculated for each sample and converted to μmol min^–1^ using 2130 M^–1^ cm^–1^ as the extinction coefficient for ONPG. Rates were normalized by total protein added to each reaction to generate a specific activity per unit total protein for each sample.

### Compound quantification

Glucose, xylose, αKIV, and fermentation products (acetate, lactate, formate) were detected using an HPLC (Agilent 1200) with an Aminex HPX-87H anion exchange column (Bio-Rad). Cell-free supernatants were loaded onto the column with an inlet and outlet temperature of 35 °C and flow rate of 0.6 ml min^–1^ under isocratic conditions with 5 mM sulfuric acid. Compound concentrations were determined by integrating the refractive index detection chromatogram and comparison to a standard curve generated with known concentrations of each commercially available standard (Sigma). Purified standards were also used to determine retention times: glucose—9 min, xylose—9.7 min, αKIV—10.2 min, lactate— 13.0 min, formate—14.3 min, and acetate—15.6 min.

Alcohols and isobutyraldehyde were detected using a gas chromatography system (Agilent 7890B GC) equipped with a VF-wax column (Agilent 30 m × 0.25 mm × 0.50 μm), flame-ionization detector (FID) and mass spectrometer (MS). All compounds were extracted into ethyl acetate from cell-free supernatants prior to analysis. The temperature program for the GC oven was 80 °C hold for 3 min, 5 °C min^–1^ ramp to 210 °C, and 210 °C hold for 3 min. Compound concentrations were determined by integrating the FID chromatogram and comparison to a standard curve generated with known concentrations of each commercially available standard (Sigma). To generate a standard curve using purified compounds, mixtures of compounds were extracted from LB or M9-based semi-defined medium using ethyl acetate, identical to the procedure for microbially generated compounds. Purified standards were also used to determine retention times, and identification of microbially produced compounds was confirmed using the MS fingerprint. The retention times for the molecules in this study are: isobutyraldehyde —4.2 min, isobutanol—7.2 min, isopentanol—8.9 min, and phenylethyl alcohol (PEA)—27.2 min. Yield of isobutanol on αKIV was calculated by dividing the moles of isobutanol produced by the moles of αKIV substrate consumed, noting a 1:1 molar relationship of aKIV conversion to isobutanol (Fig. 4a and Supplementary Table 4).

To measure microbially generated compounds found in the scCO_2_ headspace of high-pressure stainless steel columns, the headspace was de-pressurized directly into chilled ethyl acetate. The 316 stainless steel tubing and valves in a pressurization manifold^22^ enabled the simultaneous de-pressurization of 12 samples. Compounds present in the ethyl acetate were analyzed by GC, as was performed for other extracted molecules. Prior to extraction, steel tubing was flushed with ethyl acetate to eliminate cross contamination.

Isobutanol was also extracted from a custom built extractor-fermenter^12^ using scCO_2_. A 1% w/v isobutanol (Sigma) solution was prepared in deionized water and extraction was carried out at scCO_2_ flow rates of 1.3, 3.2, 5.4, and 9.0 ml min^–1^. The scCO_2_ was de-pressurized through chilled methanol to collect the isobutanol, which was quantified by GC-FID^12^. Recovery rate was determined by fitting the natural log of the fraction recovered as a function of time^12^.

### scCO_2_ fermentation-extraction process model

Aspen Plus (AspenTech) was used to simulate the isobutanol extraction process (full process: Supplementary Fig. 15a, simplified process schematic: Fig. 5a). Preliminary calculations and comparison with published data^45,46^ suggested use of the Soave-Redlich-Kwong equation of state to model phase equilibrium in all cases except for scCO_2_ extraction of isobutanol, for which our own measurements on isobutanol were used in concert with literature data on *n*-butanol separation performance^43,44^. Thermodynamic properties were modeled using the Lee-Kessler-Plöcker equation of state, as recommended by Aspen for supercritical fluids. The process feeds were CO_2_ at 0.1 MPa and a stream of aqueous glucose (media and substrate). The water feed (P1) was pumped to 10 MPa in a single stage. Consistent with typical engineering practice, CO_2_ pressurization to 10 MPa was completed in four stages (C1-4), with each stage accomplishing a threefold pressure increase. In all cases, the thermodynamic efficiency of the pumps/compressors was 85% and their mechanical efficiency was 35%. Heat exchangers (HE1-3) were used to maintain CO_2_ temperature at 40 °C after compression and for feeding the fermenter. The fermenter was simulated as consisting of a continuously fed stirred tank reactor and a separator (V2) configured in series. The reactor was modeled as achieving a steady state isobutanol concentration of 20 g l^–1^, consistent with reported titers of isobutanol^27^. The entire contents of the reactor were fed to the separator (V2), a counter-current isobutanol extraction column^44^, which separated the feed into water-rich and CO_2_-rich streams. The isobutanol composition of the exit streams were set to that reported by Laitinen and Kaunisto^44^ and modified using our performance data for isobutanol (i.e., 99.73% butanol recovery) and the exiting water and CO_2_ streams were assumed to be at mutual saturation, using the data reported by King et al.^54^ for binary mixtures. Downstream of the fermenter, a three-phase separator (V3) was used to decrease the pressure to permit the separation of isobutanol-rich, water-rich, and CO_2_-rich streams, as suggested by de Filippi and Moses^43^. Pressures tested in the three-phase separator ranged from 0.1 to 6.5 MPa, the pressure at which an isobutanol-rich phase first appears. The CO_2_-rich stream exiting the three-phase separator was either recycled or purged, with the ratio between these streams set by the splitter (V5). The CO_2_ recycle ratio was defined by the relative mass flow rates of the CO_2_ streams exiting the splitter; e.g., 50% CO_2_ recycle implies that 50% of the CO_2_ entering the splitter is recycled and 50% is off gassed. The isobutanol-rich phase was further de-pressurized to 0.1 MPa (V4), if necessary, to recover high-purity isobutanol liquid. Finally, the water exiting the three-phase separator was purged, though it could be recycled in an industrial process.

### Reporting Summary

Further information on experimental design is available in the Nature Research Reporting Summary linked to this Article.

## Supplementary Information

Supplementary Information

Source Data File

Reporting Summary

## Data Availability

The datasets generated during and/or analyzed during the current study are available from the corresponding authors on reasonable request. A Reporting Summary for this Article is available as a Supplementary Information file. The source data underlying Figs. 2, 3, 4, and 5, Supplementary Figs. 1, 3–5, 8–11, 14, and 15, Supplementary Tables 1–6 are provided as a Source Data file.
