# Supplementary material for: Engineered microbial biofuel production and recovery under supercritical carbon dioxide
Source: Nat Commun. 2019 Feb 4;10:587. doi: 10.1038/s41467-019-08486-6 (PMC6361901; doi:10.1038/s41467-019-08486-6)
Supplement: Supplementary file 1 — Supplementary Information [file 41467_2019_8486_MOESM1_ESM.pdf]

Engineered microbial biofuel production and recovery under supercritical carbon dioxide

Boock *et al.*

Supplementary Note 1. Transformation of *B. megaterium* strain SR7

Shuttle and integration plasmids have been established for strains of *B. megaterium*[1], and transformation of the species was possible through electroporation[2], protoplasting-osmotic shock[1], or conjugation[3]. Unmodified, none of these techniques led to successful transformation of SR7 in our hands. Since protoplast-osmotic shock is the most widely utilized transformation technique for other *B. megaterium* strains, this became the focus of transformation optimization efforts. SR7 cells treated with 300 µg ml^{-1} lysozyme, as prescribed for the *B. megaterium* strain DSM 319 (the most widely utilized *B. megaterium* strain), resulted in protoplasts being formed more rapidly (< 10 minutes) than is described for DSM 319[1]. Plating of SR7 protoplasted cells on non-selective medium revealed a severe loss of viable cell count due to the lysozyme treatment. Reducing the lysozyme concentration to 30 µg ml^{-1} and lowering the incubation time with lysozyme to 10 minutes improved SR7 viability by 2- to 3-fold. Coupling this lysozyme treatment with PEG-based osmotic shock led to the successful transformation of SR7 with plasmid DNA, yielding 1-5 colony forming units (CFUs) per transformation using 3 µg of DNA. Further optimization of transformation resulted from characterization of single-carbon source preference of SR7, showing high SR7 metabolic activity on L-malic acid, a TCA intermediate, and no activity by SR7 on D-malic acid[4]. Two of the buffers used in the protoplast protocol contain malic acid[1], and replacing DL-malic acid with enantiopure L-malic acid resulted in an additional 5-10 fold improvement in transformation efficiency, 10-50 CFUs per 3 µg DNA.

Supplementary Note 2. Heterologous expression of *lacZ* under 0.1 MPa CO_2

To begin assessing heterologous protein production in low pressure CO_2 environments, seed cultures under 0.1 MPa CO_2 were inoculated with endospores of SR7 that had been prepared from cultures of SR7 pXyl *lacZ*. These seed cultures were passaged into fresh semi-defined medium under 0.1 MPa CO_2, grown to exponential phase, induced with xylose and then assayed for LacZ specific activity (**Figure 3**). Similar to GFP production in aerobic cultures, β-galactosidase expression by SR7 under 0.1 MPa CO_2 with pXyl *lacZ* was 32-fold greater in xylose-induced cultures than uninduced cultures. Since *B. megaterium* contains an endogenous copy of *lacZ*, lysates of SR7 control cells containing empty plasmid were also assayed for β-galactosidase activity. Empty plasmid control cultures with and without xylose were nearly identical (**Figure 3**), and showed 8-fold less activity than uninduced pXyl *lacZ* samples, verifying that the pXyl promoter is leaky in SR7 under both anaerobic and aerobic conditions. Overall, these results indicate that the pXyl promoter is functional in anaerobic cultures at 0.1 MPa CO_2, and that SR7 maintains exogenous plasmids throughout endospore preparation as well as during an initial round of seed culturing and passaging.

Supplementary Note 3. Advantages of $scCO_2$ extraction, process model parameters for isobutanol and sensitivity analysis on process energetics

The main energy benefits of $scCO_2$ extraction are the result of it both extracting isobutanol and enabling recovery of high purity product. A competing strategy, gas stripping, removes nearly as much water as isobutanol due to the similar vapor pressures of water and the target alcohol. By comparison, $scCO_2$ extraction is much more selective for organic compounds than water due to more favorable thermodynamic partitioning, enabling recovery of isobutanol at higher concentrations[5]. A second competing technology, liquid extraction, requires distillation to recover high purity products. In contrast, recovery from $scCO_2$ occurs by simply decreasing the pressure, resulting in one-step recovery of high purity product (e.g., 94-96 wt%), which is greater than the butanol-water azeotrope[6]. Moreover, integration of fermentation and extraction, as proposed here, provides an additional energy benefit: co-production of a high-pressure CO_2 product along with isobutanol[7, 8]. CO_2 formed during fermentation is produced at 10 MPa pressure, meaning that the CO_2 product is available at system pressure to be recycled back to the fermenter, thereby reducing the amount of CO_2 that must be compressed from atmospheric pressure to 10 MPa and thus lowering energy costs.

Due to the numerous advantages outlined above for supercritical CO_2 extraction, we sought to model a combined fermentation and extraction bioprocess. Biological production using SR7 has focused on isobutanol due to its generation only requiring two enzymatic steps and its high yield. However, *n*-butanol is a more widely studied compound in terms of thermodynamics[9, 10] and extraction from $scCO_2$ systems[5, 11, 12]. For physical property estimation, we used the Lee-Kesler-Plocher equation of state, as recommended by the software. For phase behavior analysis, we used isobutanol thermodynamic properties and the Soave-Redlich-Kwong equation of state, except for the extractor. In the extractor, we used data reported for *n*-butanol extraction efficiency[11], and modified it using our own *n*-butanol and isobutanol extraction measurements. Quantitatively, literature data report 99.7% *n*-butanol recovery for $scCO_2$ extraction in a supercritical fluid-liquid extraction column operating at 10 MPa and 40 °C and at 3:1 $scCO_2$-to-butanol feed ratio[11]. Using our own comparative data, which indicated superior isobutanol extraction performance compared to *n*-butanol (**Supplementary Figure 13**), the recovery was adjusted to 99.73%.

The CO_2:isobutanol feed ratio is a crucial variable for process energy efficiency, with the energy demand increasing linearly with CO_2:isobutanol ratio (**Supplementary Figure 15b**), largely due to CO_2 compression being the greatest energy cost in the process. CO_2:isobutanol ratios are determined by desired purity, partition coefficient and extractor configuration. For our model and based on literature reports, CO_2:isobutanol ratios less than 3:1 are included (**Supplementary Figure 15b**) – still maintaining 99.73% isobutanol recovery – to show the benefits of improving extraction efficiency to levels greater than those reported[13]. In contrast, CO_2:isobutanol ratios greater than 3:1 show the penalty associated with less efficient extraction; however, even for a 6:1 CO_2:isobutanol ratio, the energy efficiency of isobutanol extraction remains attractive (5-7 MJ kg^{-1}).

In addition to minimizing the CO_2:isobutanol ratio, process efficiency can be improved by two-stage de-pressurization of the CO_2-butanol mixture. First, the mixture is de-pressurized to a pressure intermediate to fermentation (10 MPa) and atmospheric (0.1 MPa) to generate a three phase mixture consisting of an isobutanol-rich phase with low water content, a water-rich aqueous phase, and a CO_2-rich vapor phase that can be recycled. Second, de-pressurization to 0.1 MPa to allows recovery of the isobutanol product. In all cases, the highest pressure that allowed for an isobutanol-rich phase to occur, 6.5 MPa, was found to be energetically optimal. For CO_2:isobutanol feed ratios greater than 6:1, optimizing the intermediate pressure can decrease energy requirements by as much as 3 MJ kg^{-1} (**Supplementary Figure 15b**). For CO_2:isobutanol ratio less than 6:1, the effect of varying the intermediate CO_2 pressure was found to be less than 1.0 MJ kg^{-1} (**Supplementary Figure 15b**). Nonetheless, the 1.0 MJ kg^{-1} difference may be significant as a percentage of the overall energy requirement, which is less than 6.0 MJ kg^{-1}, suggesting intermediate pressure is a variable to evaluate in practice.

Lastly, the effect of CO_2 recycle ratio was assessed at the optimal intermediate pressure of 6.5 MPa and for a CO_2:isobutanol feed ratio of 3:1. CO_2 recycle ratio is defined as the ratio of CO_2 by mass that is returned to the compression stages relative to the total exiting the decompression vessel in the gas/vapor phase stream (**Supplementary Figure 15a,c**). Energy requirements decrease monotonically with increasing recycle ratio, consistent with increasing recycle ratio decreasing the amount of CO_2 that must be pressurized from 0.1 to 10 MPa. The maximum CO_2 recycle ratio is approximately 87%; because of co-generation of CO_2 during fermentation of glucose to isobutanol, increasing the recycle ratio to values greater than 87% results in CO_2 accumulation, which is not desired.

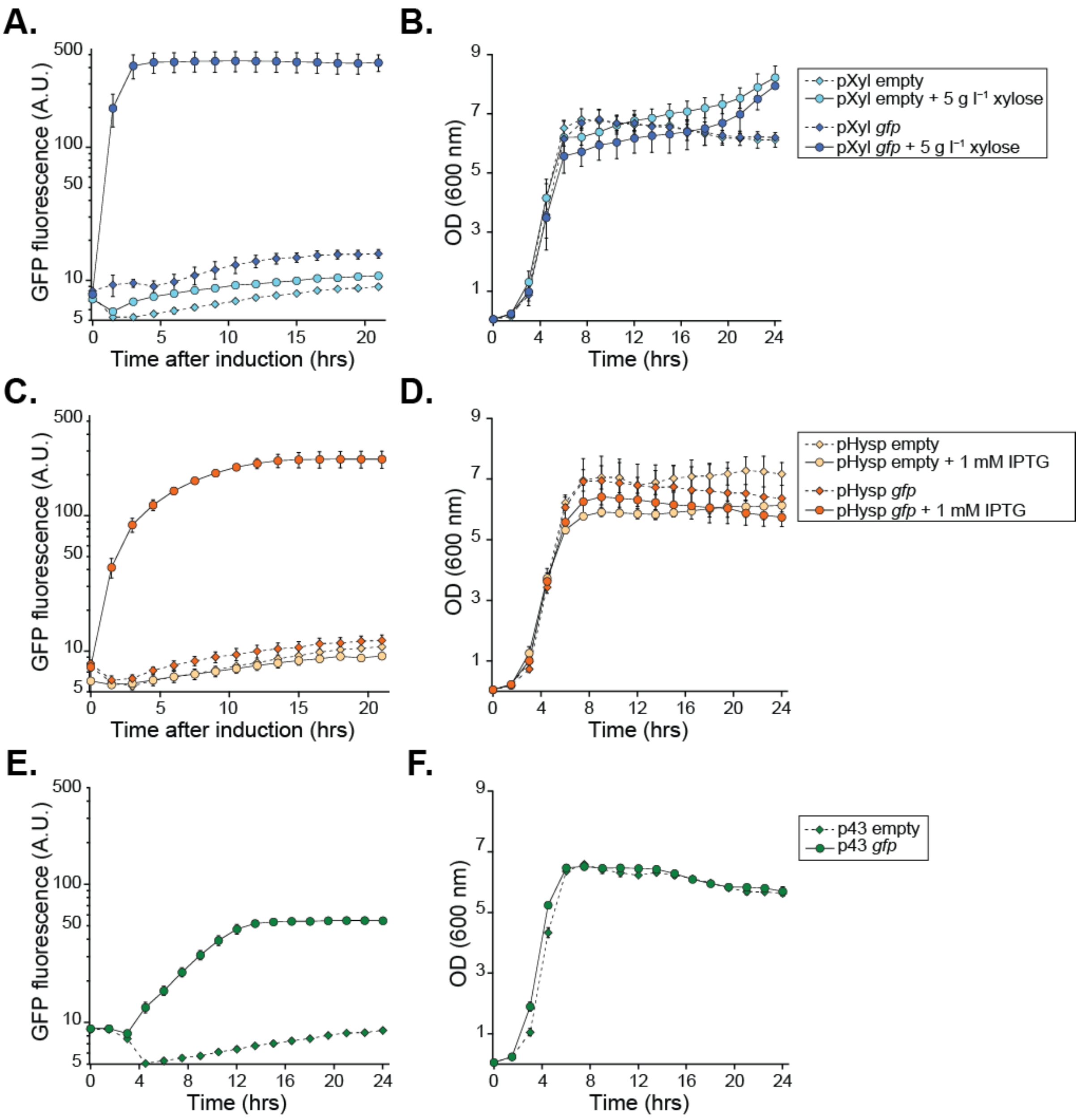

Supplementary Figure 1. Bulk fluorescence of SR7 containing *gfp*-encoded plasmids grown aerobically in LB medium. (**a**) GFP fluorescence and (**b**) optical density (measured at 600 nm) of a population of SR7 cells containing pXyl empty (light blue) or pXyl *gfp* (dark blue). Diamonds with dashed lines show unamended cultures, and filled circles with solid lines show those with xylose added to 5 g l^{-1} at 3 hours of culture time. (**c**) GFP fluorescence and (**d**) optical density of a population of SR7 cells containing pHysp empty (light orange) or pHysp *gfp* (dark orange). Diamonds with dashed lines show uninduced cultures, and filled circles with solid lines show samples with IPTG added to 1 mM at 3 hours of culture time. (**e**) GFP fluorescence and (**f**) optical density of a population of SR7 cells containing p43 empty (green diamonds with dashed lines) or p43 *gfp* (closed green circles with solid lines). For all measurements, error bars represent the standard deviation of biological triplicate samples. Source data are provided as a Source Data file.

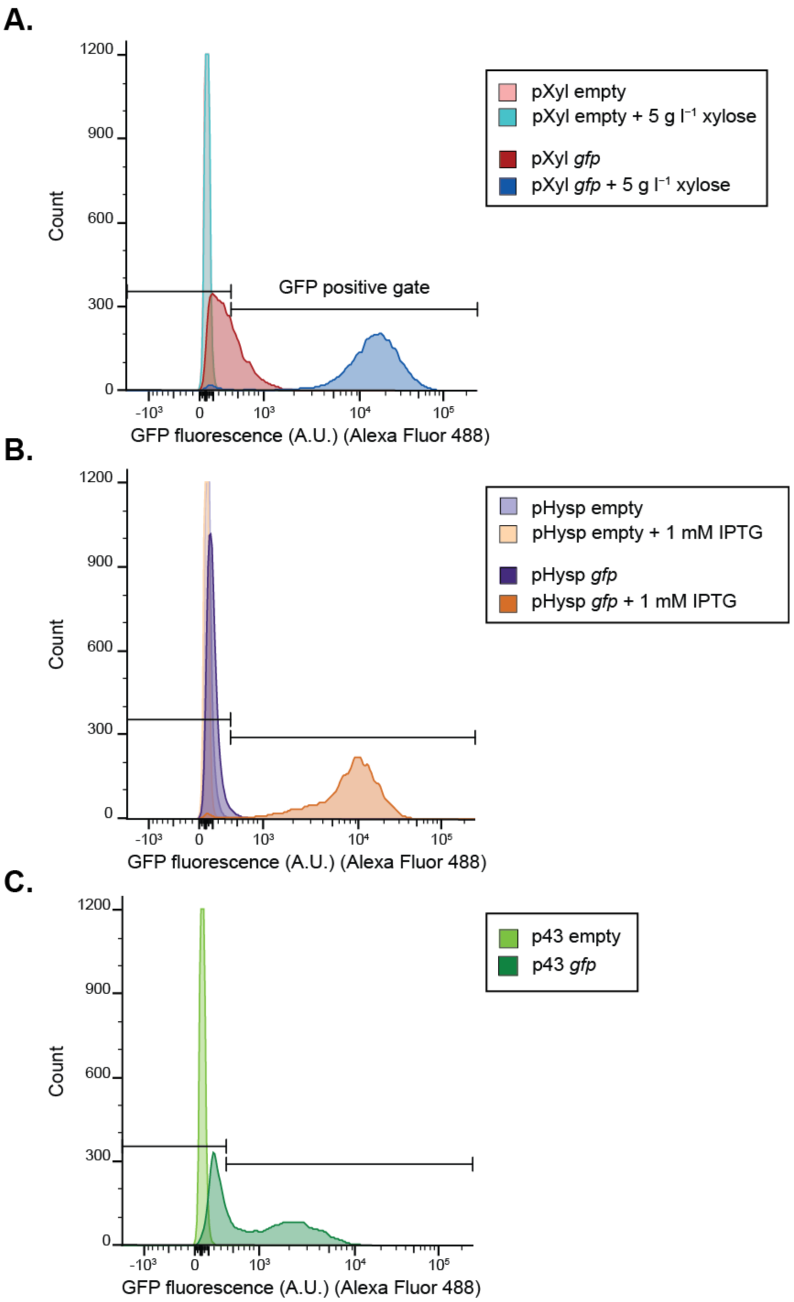

Supplementary Figure 2. Fluorescence populations of SR7 cells expressing *gfp* measured by FACS. Representative GFP-fluorescence distribution histograms of a population of SR7 cells containing plasmids (**a**) pXyl, (**b**) pHysp, or (**c**) p43. Controls without *gfp* and without inducer are shown for each plasmid set. Cultures were grown aerobically in LB medium and FACS analysis was performed 4 hours post induction for (a) and (b), and at 7 hours total culture time for (c). Induction with 5 g l^{-1} xylose (a) or 1 mM IPTG (b) occurred after 3 hours of culturing. GFP positive gate was established by gating above the autofluorescence observed for all empty plasmid containing controls. All samples were performed in biological triplicate and only the first sample of the set is shown here for clarity. GFP positive gating of each population set is found in Supplementary Table 1. Source data are provided as a Source Data file.

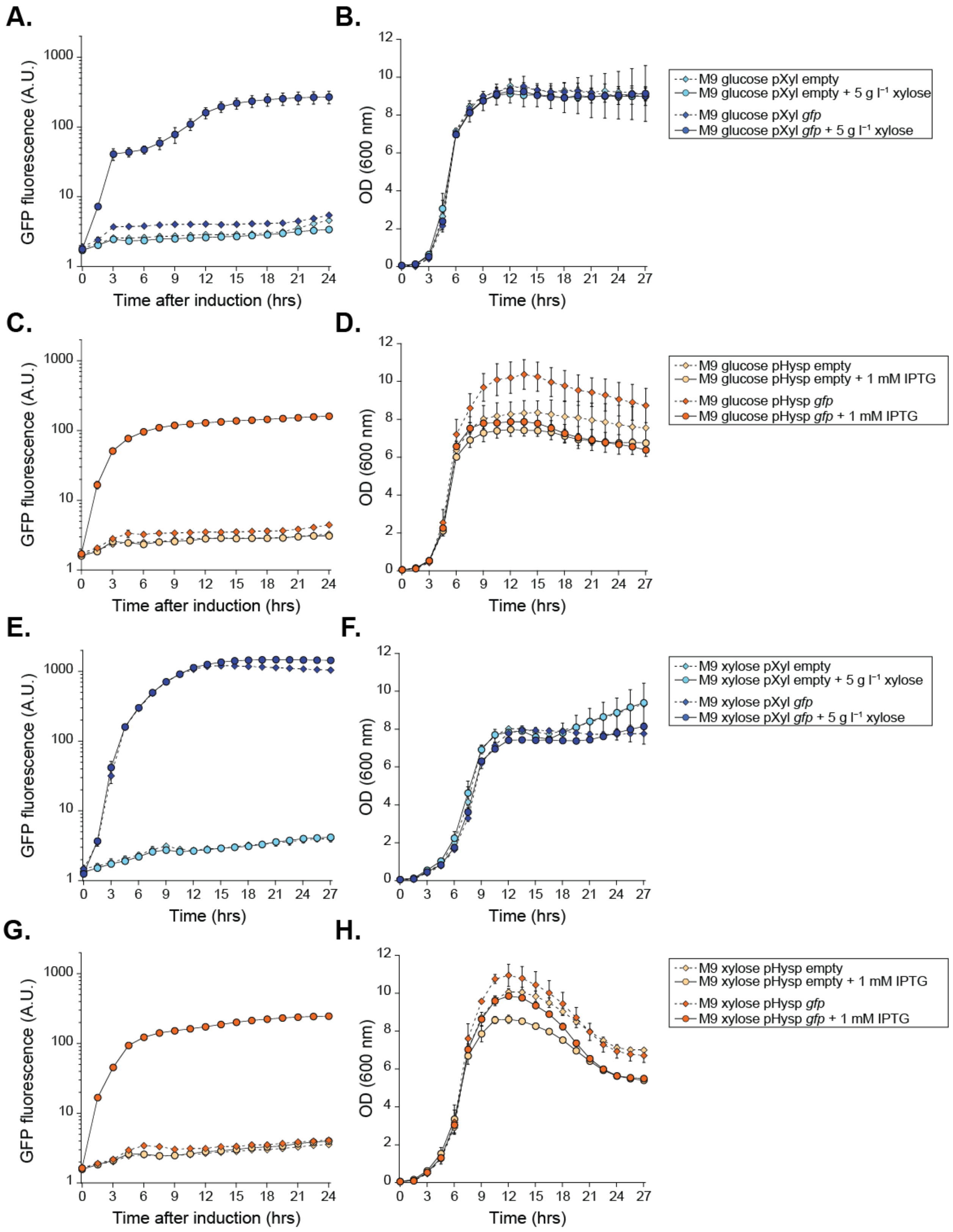

Supplementary Figure 3. Bulk fluorescence of SR7 containing *gfp*-encoded plasmids grown aerobically in M9-based semi-defined medium.

(**a**) GFP fluorescence and (**b**) optical density (measured at 600 nm) of a population of SR7 cells containing pXyl empty (light blue) or pXyl *gfp* (dark blue) grown in semi-defined medium with 10 g l^{-1} glucose. Diamonds with dashed lines show unamended cultures, and filled circles with solid lines show those induced with 5 g l^{-1} xylose at 3 hours of culture time (OD ~ 0.4). (**c**) GFP fluorescence and (**d**) optical density of a population of SR7 cells containing pHysp empty (light orange) or pHysp *gfp* (dark orange) grown in semi-defined medium with 10 g l^{-1} glucose. Diamonds with dashed lines show uninduced cultures, and filled circles with solid lines show samples induced with 1 mM IPTG at 3 hours of culture time. (**e**) GFP fluorescence and (**f**) optical density of a population of SR7 cells containing pXyl empty (light blue) or pXyl *gfp* (dark blue) grown in semi-defined medium with 10 g l^{-1} xylose. Diamonds with dashed lines show unamended cultures, and filled circles with solid lines show those induced with 5 g l^{-1} xylose at 3 hours of culture time. Note for (e), total culture time is provided since xylose is both a carbon source and inducer. (**g**) GFP fluorescence and (**h**) optical density of a population of SR7 cells containing pHysp empty (light orange) or pHysp *gfp* (dark orange) grown in semi-defined medium with 10 g l^{-1} xylose. Diamonds with dashed lines show uninduced cultures, and filled circles with solid lines show samples induced with 1 mM IPTG at 3 hours of culture time. In addition to 10 g l^{-1} glucose or xylose, semi-defined medium contained 1x M9 salts, 0.05 g l^{-1} yeast extract, 5 μg ml^{-1} tetracycline and 1x trace metals supplement. For all measurements, error bars represent the standard deviation of biological triplicate samples. Source data are provided as a Source Data file.

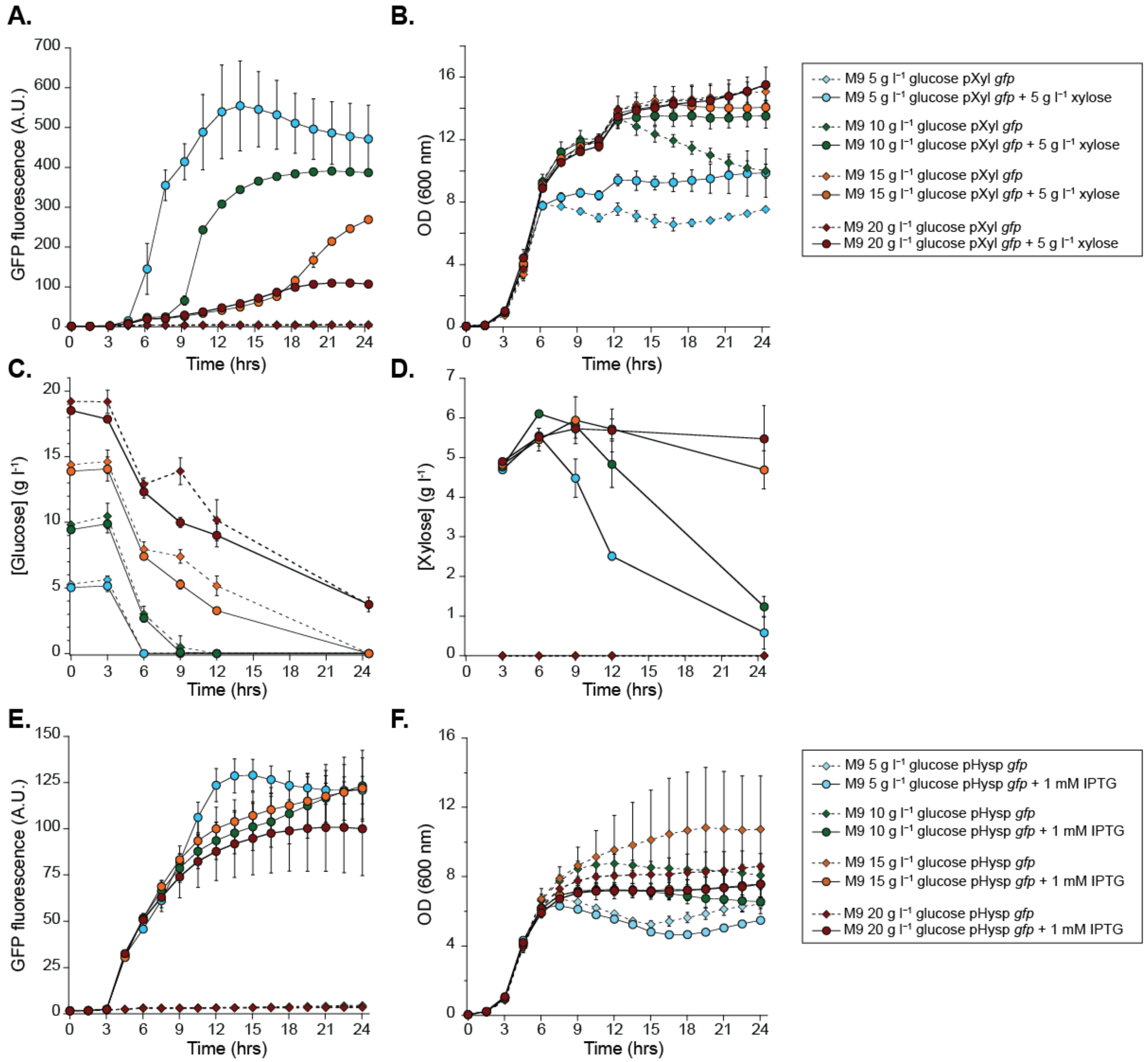

Supplementary Figure 4. Bulk fluorescence of SR7 containing *gfp*-encoded plasmids grown aerobically in M9-based semi-defined medium with various concentrations glucose. (**a**) GFP fluorescence, (**b**) optical density (measured at 600 nm), (**c**) glucose concentration, and (**d**) xylose concentration of a population of SR7 cells containing pXyl *gfp* grown in semi-defined medium with 5 g l^{-1} glucose (light blue), 10 g l^{-1} glucose (dark green), 15 g l^{-1} glucose (dark orange), or 20 g l^{-1} glucose (dark red) under aerobic conditions. Diamonds with dashed lines show uninduced cultures, and filled circles with solid lines show samples induced with 5 g l^{-1} xylose at 3 hours of culture time. In addition to glucose, semi-defined medium contained 1x M9 salts, 0.05 g l^{-1} yeast extract, 5 µg ml^{-1} tetracycline, and 1x trace metals supplement. (**e**) GFP fluorescence and (**f**) optical density of a population of SR7 cells containing pHysp *gfp* grown in semi-defined medium with 5 g l^{-1} glucose (light blue), 10 g l^{-1} glucose (dark green), 15 g l^{-1} glucose (dark orange), or 20 g l^{-1} glucose (dark red) under aerobic conditions. Diamonds with dashed lines show uninduced cultures, and filled circles with solid lines show samples induced with 1 mM IPTG at 3 hours of culture time. For all measurements, error bars represent the standard deviation of biological triplicate samples. Source data are provided as a Source Data file.

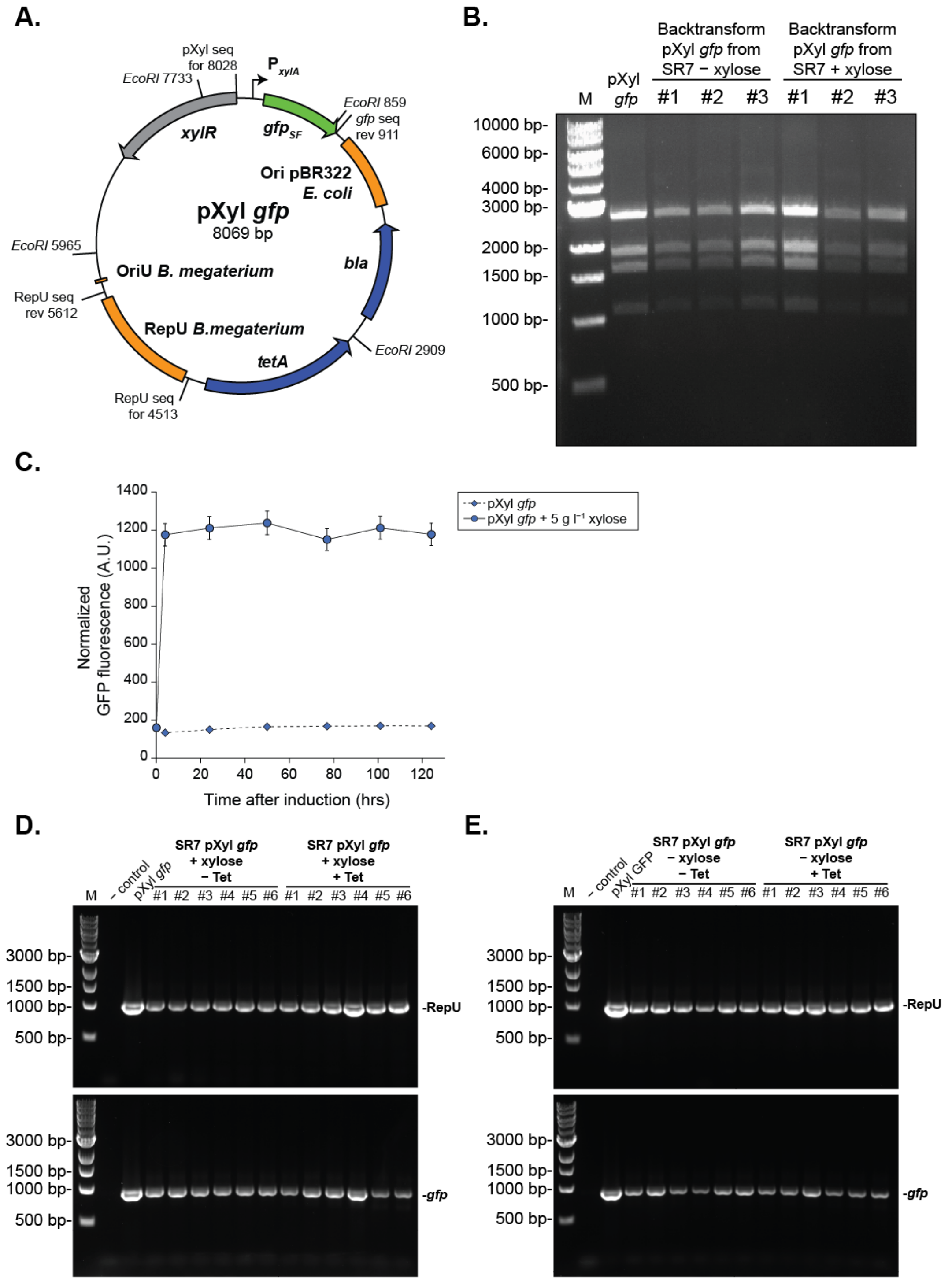

Supplementary Figure 5. Plasmid stability assessment for SR7 containing pXyl *gfp*. (**a**) Plasmid map of pXyl *gfp*, including origins of replication (orange), antibiotic resistance genes (blue), *gfp* gene (green) and *xylR* regulator (grey). Also provided are locations of *EcoRI* restriction sites and binding sites for PCR primers used for colony-PCR-based plasmid verification. (**b**) Restriction enzyme digest using *EcoRI* of pXyl *gfp* that was isolated from SR7 and backtransformed into *E. coli* for analysis. The pXyl *gfp* lane is digested plasmid DNA

isolated directly from *E. coli*. For backtransformed samples, after 24 hours of culturing SR7 pXyl *gfp* with 5 g l^{-1} xylose (+ xylose) or without xylose (– xylose) aerobically in LB medium, the pXyl plasmid was isolated from SR7 genomic DNA and backtransformed into *E. coli* DH5α, which were plated on carbenicillin-containing solid medium plates. Three colonies of DH5α were selected for each sample, and pXyl *gfp* DNA was isolated for each prior to digestion with *EcoRI*. (**c**) OD normalized fluorescence (ex: 485 nm, em: 528 nm) for SR7 pXyl *gfp* with and without 5 g l^{-1} xylose measured over 128 hours aerobically in LB medium. Prior to reading fluorescence values, OD measurements were obtained, and cultures were diluted using LB medium to a uniform OD. Error bars represent the standard deviation of biological triplicate cultures. (**d-e**) Gel electrophoresis of PCR samples of colonies of SR7 pXyl *gfp* that have been grown for 74 hours aerobically in LB medium in the presence (d) (+ xylose) and absence (e) (– xylose) of 5 g l^{-1} xylose, and have been plated on LB agar with (+ Tet) and without (– Tet) 5 μg ml^{-1} tetracycline antibiotic. To amplify the RepU fragment (1099 bp), RepU for and RepU rev primers were used. To specifically detect the *gfp* fragment (952 bp), pXyl for and GFP rev primers were used. Negative control lanes show PCR samples that did not contain any template DNA, and pXyl *gfp* lanes are PCR samples that used pXyl *gfp* isolated from *E. coli* as a template. Colony forming units measured for cultures used in (d – culture #2) and (e – culture #1) are found in Supplementary Table 2. Source data are provided as a Source Data file.

A.

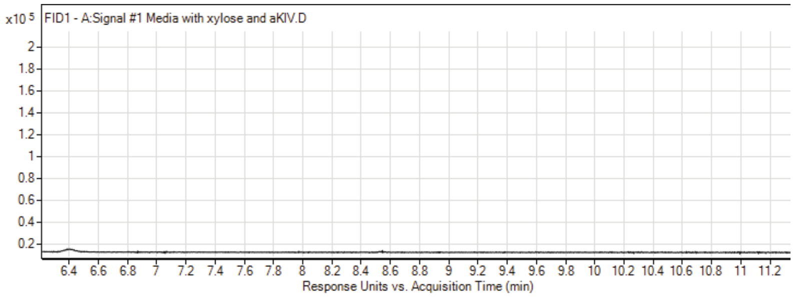

B.

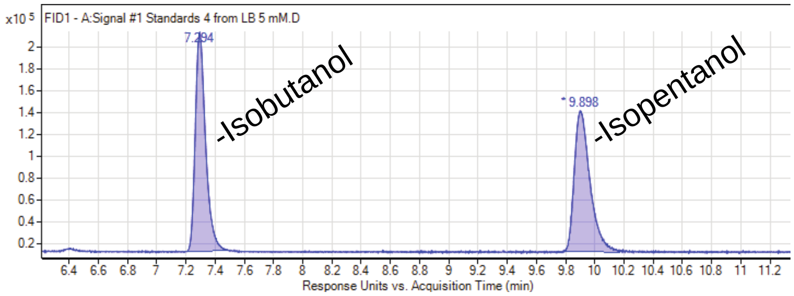

Supplementary Figure 6. GC chromatograms of isobutanol and isopentanol standards. FID chromatograms showing the separation and retention time of (**a**) LB medium with 5 g l^{-1} xylose and 5 mM αKIV and (**b**) LB medium with 5 mM isobutanol and isopentanol. For each sample, desired concentrations of standard are added to LB medium and extracted with ethyl acetate prior to separation by GC and analysis by FID. The isobutanol and isopentanol peaks are indicated in (b).

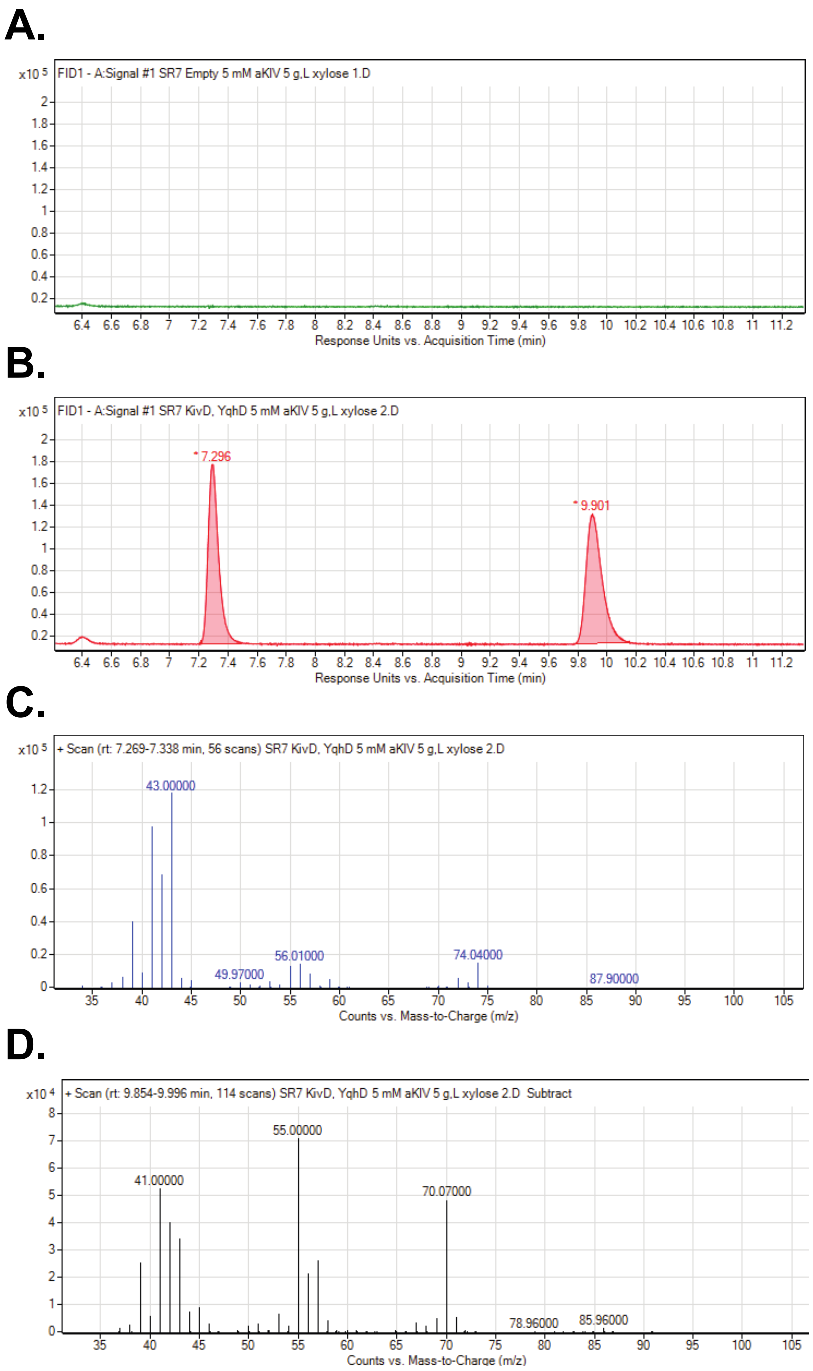

Supplementary Figure 7. GC/MS chromatograms of SR7 produced isobutanol and isopentanol. FID chromatograms of ethyl acetate extracted samples from (**a**) SR7 pXyl empty or (**b**) pXyl *kivD* $yqhD_{Ec}$ when grown aerobically in LB medium with 5 g l^{-1} xylose and 5 mM αKIV. MS fingerprint of the microbially-produced (**c**) isobutanol or (**d**) isopentanol peaks, with SR7 pXyl empty subtracted from pXyl *kivD* $yqhD_{Ec}$.

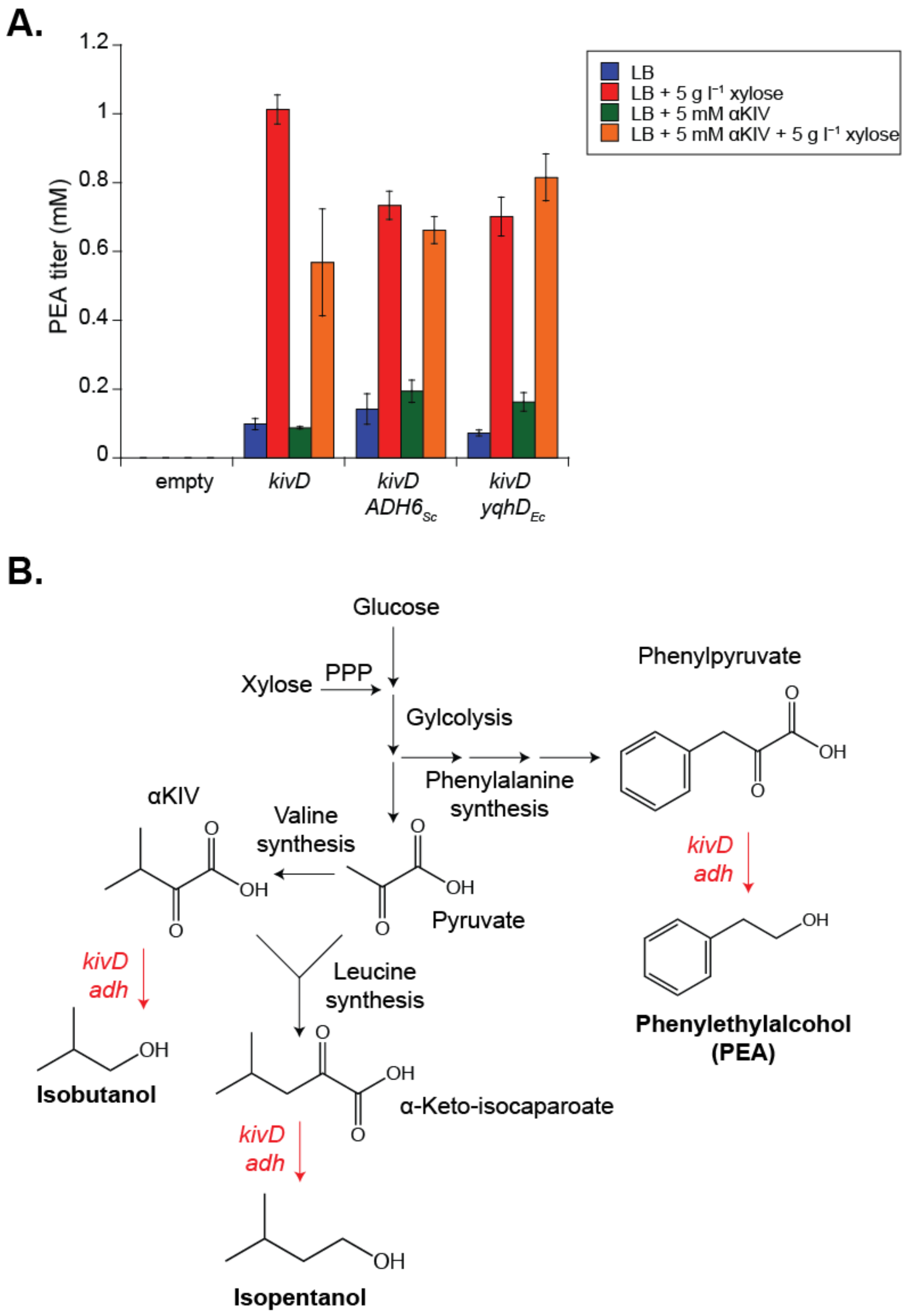

Supplementary Figure 8. Phenylethylalcohol (PEA) synthesis for SR7 strains that express *kivD* and predicted pathways to generate higher alcohols. (**a**) Production of PEA in SR7 that contain an empty pXyl plasmid, pXyl *kivD*, pXyl *kivD* $ADH6_{Sc}$ or pXyl *kivD* $yqhD_{Ec}$. Cultures were grown aerobically in LB medium for 24 hours with and without 5 g l^{-1} xylose and 5 mM αKIV. (**b**) Predicted metabolic pathways in SR7 that could generate the alcohols observed in this work when combined with an α-ketoisovalerate decarboxylase (*kivD*) and a native or exogenous alcohol dehydrogenase (*adh*). This figure is partially adapted from Atsumi, S. et al (2008)[8]. Source data of Supplementary Figure 8A are provided as a Source Data file.

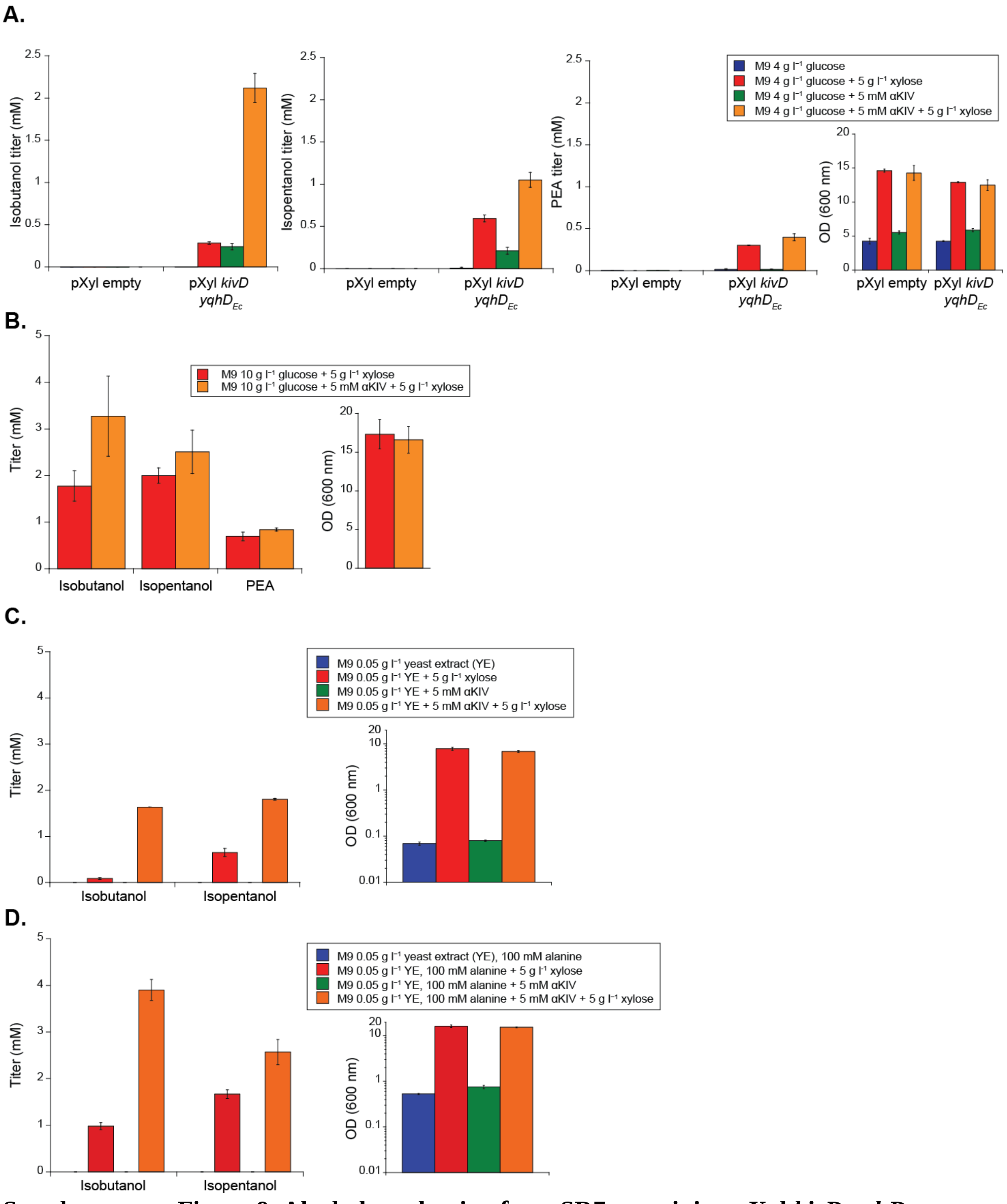

Supplementary Figure 9. Alcohol production from SR7 containing pXyl *kivD yqhD*$_{Ec}$ grown in semi-defined media aerobically. (**a**) Isobutanol, isopentanol, and PEA titers for SR7 pXyl empty or pXyl *kivD yqhD*$_{Ec}$. Cultures were grown aerobically in semi-defined minimal medium containing 1x M9 salts, 4 g l^{-1} glucose, 0.05 g l^{-1} yeast extract (YE), 5 μg ml^{-1} tetracycline, and 1x trace metal solution. Enzyme expression was induced with 5 g l^{-1} xylose and 5 mM αKIV served as a substrate for the isobutanol pathway. Production was measured 24 hours post

induction. Error bars for all samples represent the standard deviation of biological triplicate cultures. OD readings at 24 hours are also provided. (**b**) Isobutanol, isopentanol, and PEA titers at 24 hours post induction for SR7 pXyl *kivD* $yqhD_{Ec}$ when grown aerobically in semi-defined medium with an increased concentration of glucose: 1x M9 salts, 10 g l^{-1} glucose, 0.05 g l^{-1} YE, 5 µg ml^{-1} tetracycline, and 1x trace metal solution. For samples with substrate, 5 mM αKIV was used. OD readings at 24 hours are also provided. (**c**) Isobutanol and isopentanol titers at 24 hours post induction for SR7 pXyl *kivD* $yqhD_{Ec}$ when grown aerobically in semi-defined medium lacking glucose, containing only 1x M9 salts, 0.05 g l^{-1} YE, 5 µg ml^{-1} tetracycline, and 1x trace metal solution. For induced samples, 5 g l^{-1} xylose was added 2.5 hours after subculturing, regardless of OD at that time. For samples with substrate, 5 mM αKIV was used. OD readings at 24 hours are also provided (log_{10} scale). (**d**) Isobutanol and isopentanol titers at 24 hours post induction for SR7 pXyl *kivD* $yqhD_{Ec}$ when grown aerobically in semi-defined medium lacking glucose with 100 mM alanine. The medium used for these experiments contained 1x M9 salts, 0.05 g l^{-1} YE, 5 µg ml^{-1} tetracycline, 1x trace metal solution, and 100 mM alanine. For induced samples, 5 g l^{-1} xylose was added 2.5 hours after subculturing, regardless of OD at that time. For samples with substrate, 5 mM αKIV was used. OD readings at 24 hours are also provided (log_{10} scale). Source data are provided as a Source Data file.

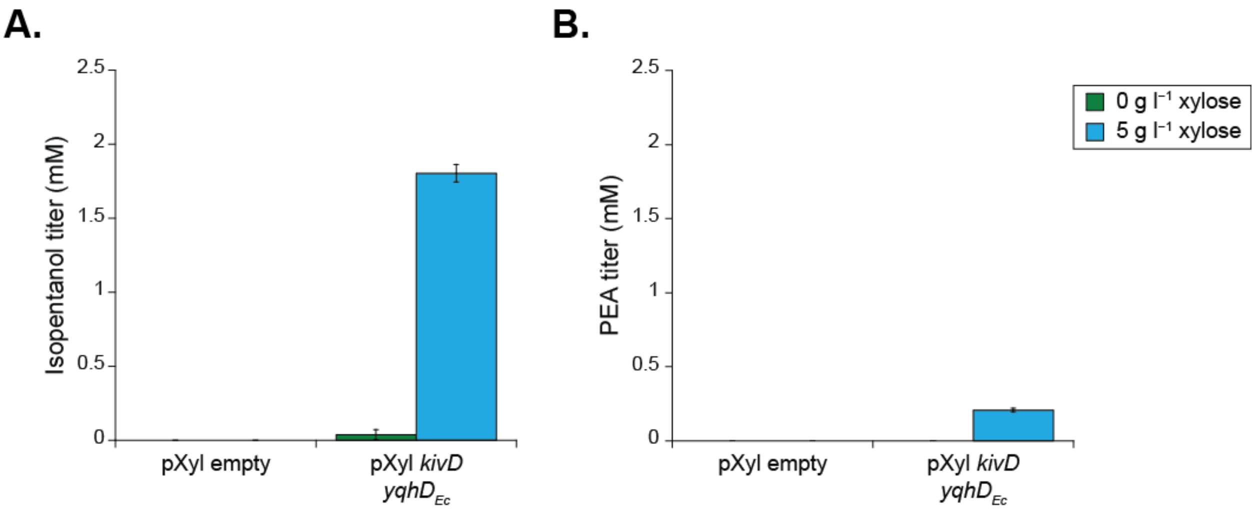

Supplementary Figure 10. Isopentanol and PEA titers for SR7 pXyl *kivD yqhD$_{Ec}$* grown in semi-defined medium under 0.1 MPa CO_2. (**a**) Isopentanol and (**b**) PEA titers for 48 hour cultures of SR7 grown under 0.1 MPa CO_2 in semi-defined medium. Semi-defined medium contains 1x M9 salts, 4 g l^{-1} glucose, 0.05 g l^{-1} YE, 0.5 µg ml^{-1} tetracycline, and 0.1x trace metal solution. Induction was initiated using 5 g l^{-1} xylose, and 5 mM αKIV served as the substrate for the isobutanol pathway. Error bars represent the standard deviation of quadruplicate cultures. Source data are provided as a Source Data file.

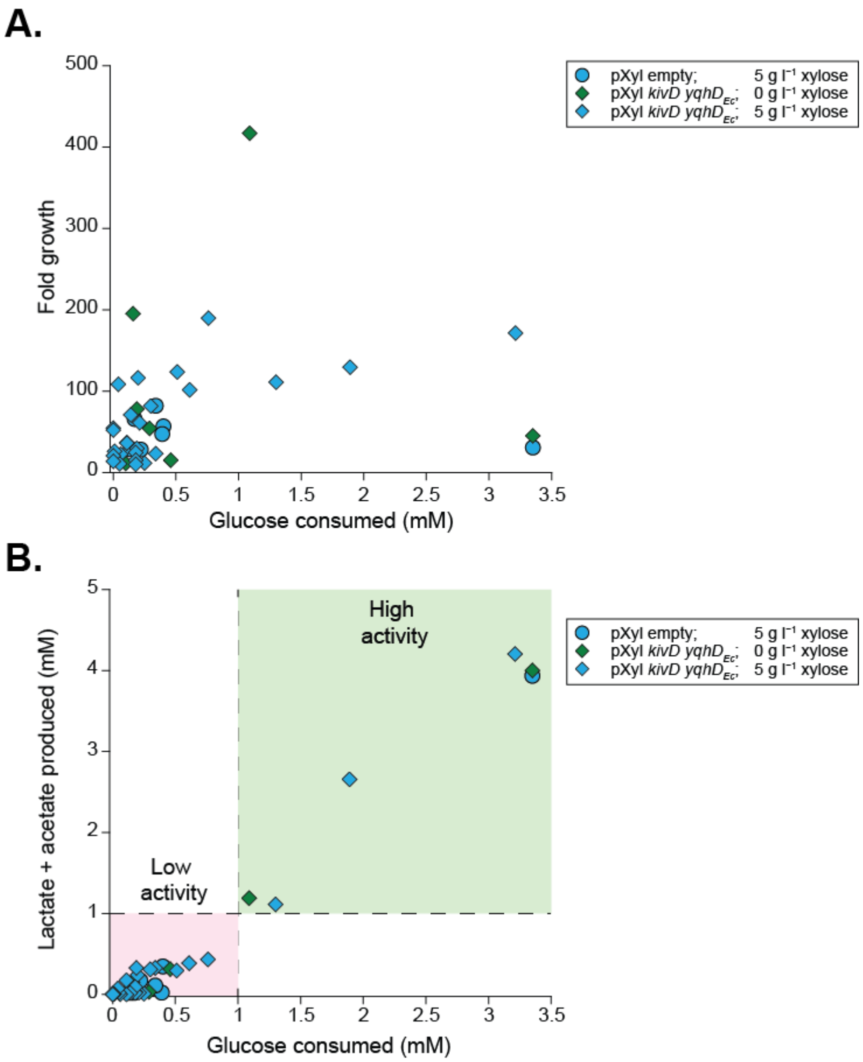

Supplementary Figure 11. Fold growth and fermentation product generation as a function of glucose consumption for SR7 cultures grown under scCO$_2$. (**a**) Fold growth and (**b**) titer of combined lactate and acetate fermentation products as a function of glucose consumed by SR7 pXyl empty or pXyl *kivD yqhD*$_{Ec}$ cultures grown under scCO$_2$ for 21 days in semi-defined medium. For all cultures, the semi-defined medium contained 1x M9 salts, 0.05 g l^{-1} yeast extract, 0.1x trace metals supplement, 4 g l^{-1} glucose (22.2 mM glucose), 100 mM L-alanine, and 5 mM αKIV. For induced cultures, 5 g l^{-1} xylose was also added at inoculation. Endospores of SR7 pXyl empty or pXyl *kivD yqhD*$_{Ec}$ were used to inoculate the cultures prior to pressurization to 10 MPa with scCO$_2$ in the headspace. Fold growth was determined by microscopy and comparing the inoculated endospore count to the number of vegetative cells observed after growth. Glucose consumption and fermentation product generation was measured using liquid chromatography for those samples that showed at least a 10-fold increase in cell number. Supplementary Table 3 provides the fraction of samples that showed at least a 10-fold increase in cell number. In (b), high activity cultures (shown by the box in light green) are defined as those samples that consumed at least 1 mM glucose and produced more than 1 mM combined lactate and acetate. Source data are provided as a Source Data file.

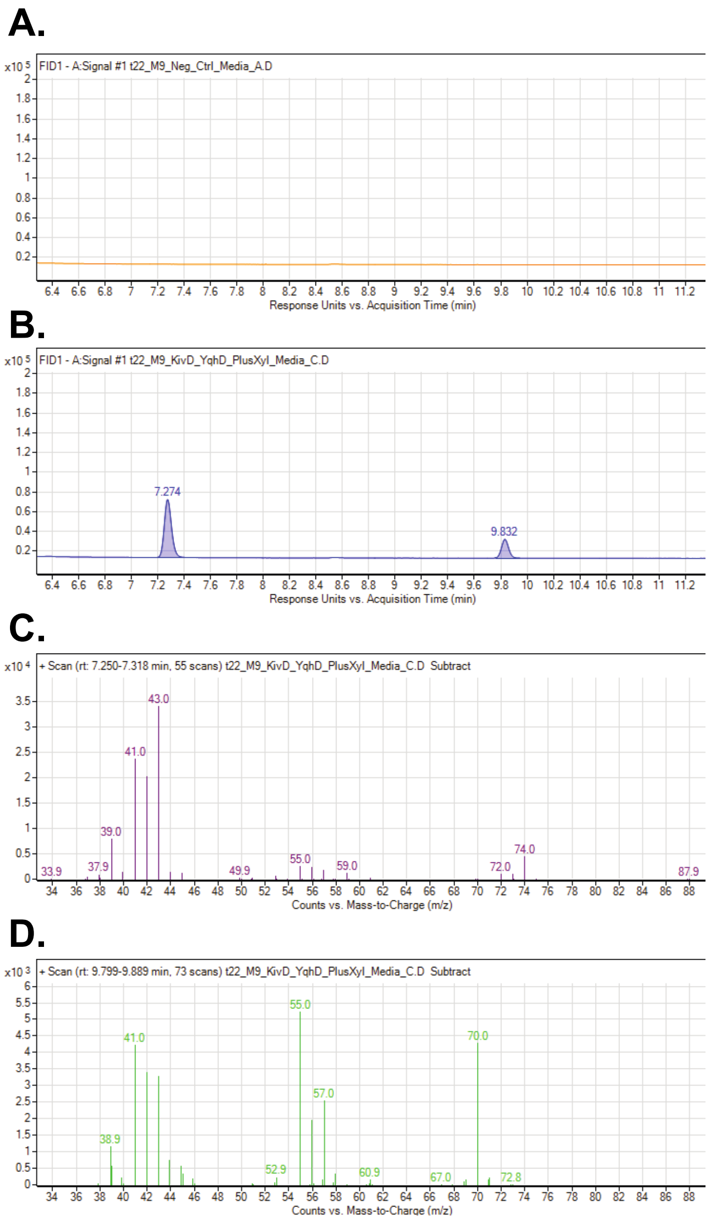

Supplementary Figure 12. GC/MS chromatograms of SR7 produced isobutanol and isopentanol extracted from the aqueous phase of cultures grown under scCO$_2$. FID chromatrograms of (**a**) negative control semi-defined medium sample with 5 g l^{-1} xylose and 5 mM αKIV, and (**b**) SR7 pXyl *kivD yqhD*$_{Ec}$ (replicate A) sample, cultured with 5 g l^{-1} xylose and 5 mM αKIV. MS fingerprint of the microbially-produced (**c**) isobutanol or (**d**) isopentanol peaks for cultures grown under scCO$_2$, with semi-defined medium subtracted from pXyl *kivD yqhD*$_{Ec}$.

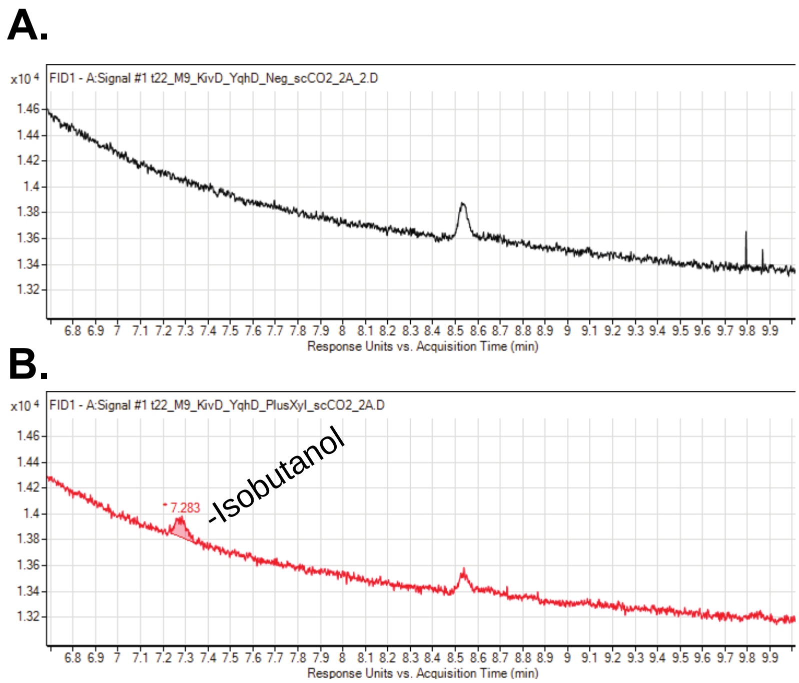

Supplementary Figure 13. GC chromatograms of SR7 produced isobutanol sampled from the headspace of cultures grown under scCO$_2$. FID chromatograms of samples extracted from the scCO$_2$ headspace above (**a**) semi-defined medium with 5 g l^{-1} xylose and 5 mM αKIV and (**b**) cultures of SR7 pXyl *kivD yqhD$_{Ec}$* in semi-defined medium with 5 g l^{-1} xylose and 5 mM αKIV. This isobutanol peak is labeled for clarity and has identical retention times as other samples displayed in **Supplementary Figures 6-7, 9-10, and 12**. For (b), 12 samples were simultaneously collected due to the design of the depressurization apparatus. Due to the low concentration of isobutanol, as shown in the FID chromatogram, a peak was not observed in the MS trace and a MS fingerprint could not be determined.

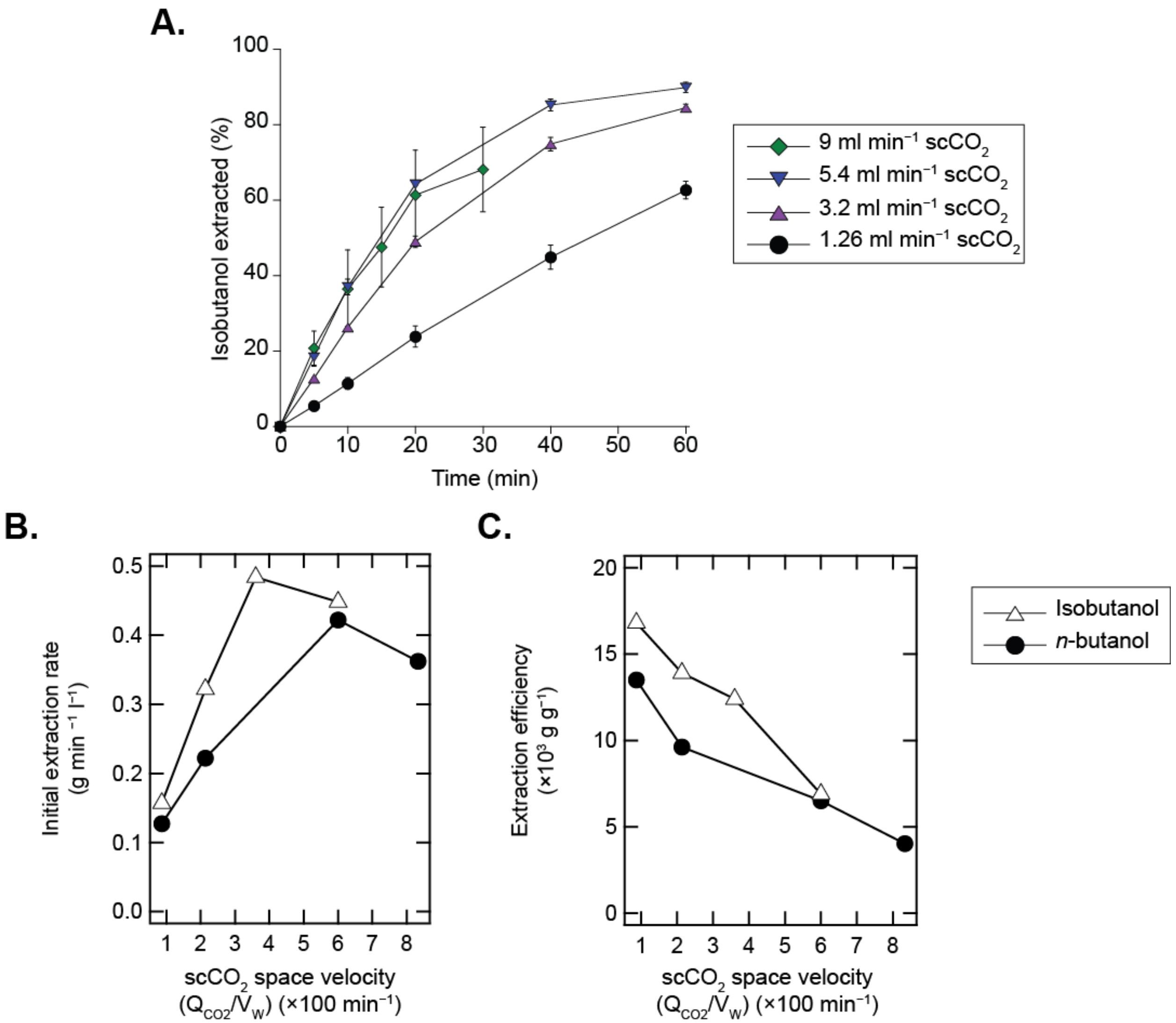

Supplementary Figure 14. $scCO_2$ extraction of isobutanol using an extractor-fermenter. (**a**) Cumulative extraction of 1% w/v isobutanol from an aqueous solution using $scCO_2$ at various volumetric flow rates: 9 ml min^{-1} (green diamond), 5.4 ml min^{-1} (blue triangle), 3.2 ml min^{-1} (purple triangle), and 1.26 ml min^{-1} (black circle). Each data point represents the average of at least two trials and error bars show the standard deviation. (**b**) Extraction rate as a function of $scCO_2$ space velocity for isobutanol (open triangle) and *n*-butanol (closed circle); *n*-butanol data is provided for comparison and is from Tompsett, G.A. et al. (2018)[5]. To calculate the extraction rate, the raw data presented in (a) was fit using a first order extraction equation (ln C/C_o), and the extraction rate is calculated from the fit. For a given $scCO_2$ flow rate (Q_{scCO2}), the space velocity is calculated by dividing Q_{scCO2} by the volume of the aqueous phase (V_W). (**c**) The extraction efficiency as a function of $scCO_2$ space velocity for isobutanol (open triangle) and *n*-butanol (closed circle). The extraction efficiency is calculated as the mass of alcohol recovered divided by the mass of $scCO_2$ required to obtain it. For comparison of different $scCO_2$ space velocities, 50% recovery of the alcohol was selected as the amount of biofuel to be recovered and the mass of $scCO_2$ required was calculated using the first order rate of extraction equation. Source data are provided as a Source Data file.

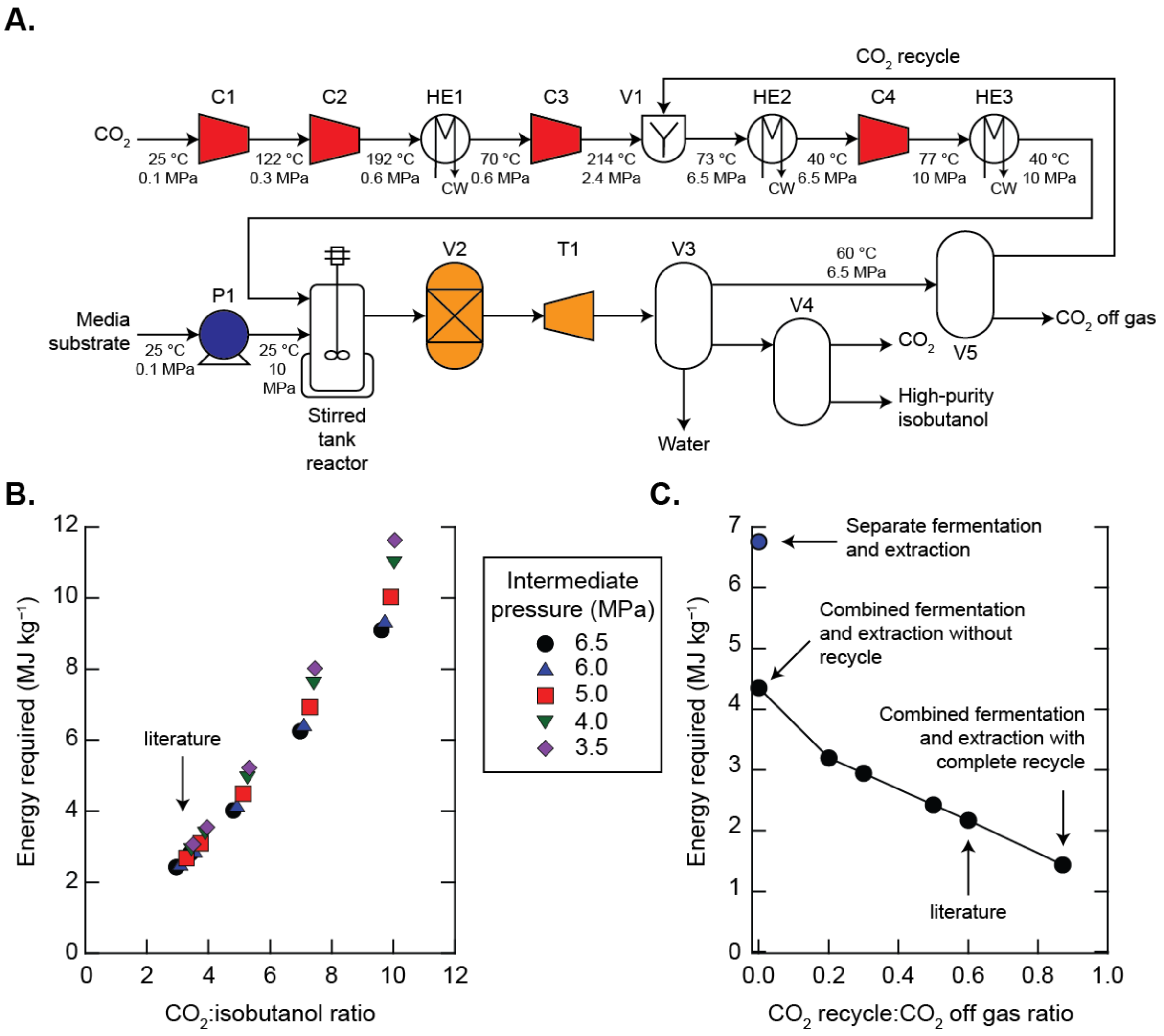

Supplementary Figure 15. Energetics of continuous $scCO_2$-based extraction of biofuels. (**a**) Full process flow diagram used for Aspen Plus simulation of continuous $scCO_2$-based extraction of microbially produced isobutanol to estimate the energetics of separation. Shown in the model are compressors (C) to pressurize CO_2 from atmospheric to 10 MPa, heat exchangers (HE) to maintain the inlet temperature of $scCO_2$ into the reactor at 25 °C, a liquid pump (P) for media and substrate, an extractor fermenter, a turbine (T) to recover energy during depressurization, and separation vessels (V) to recover high purity biofuels. This model enabled the study of 1) the effect of CO_2:isobutanol feed ratio, 2) the effect of three-phase separator pressure (intermediate pressure), and 3) the effect of CO_2 recycle ratio on overall energetic efficiency of $scCO_2$-based extraction. **(b)** Overall process energy as a function of CO_2:isobutanol feed ratio (by mass) for a fixed CO_2 recycle of 50%. A 3:1 CO_2:isobutanol feed ratio was suggested by Laitinen and Kaunisto[11] for *n*-butanol separation (literature). CO_2:isobutanol feed ratios greater than 3:1 are provided to show the effect of less efficient separation. Depressurizing to a level intermediate to fermentation pressure and atmospheric can be used to form an isobutanol-rich phase that can be separated by decantation. Moreover, partial de-pressurization makes available a partially pressurized CO_2 stream that can be recycled to the fermenter, thereby decreasing the quantity of CO_2 that must be pressurized from atmospheric to 10 MPa. For each CO_2:isobutanol feed ratio,

several intermediate pressures were evaluated: 3.5 MPa (purple diamond), 4.0 MPa (green triangle), 5.0 MPa (red square), 6.0 MPa (blue triangle), and 6.5 MPa (black circle). The effect of the intermediate pressure is modest for CO_2-to-isobutanol feed ratios less than 6:1; nonetheless, decreasing the energy requirement by 0.3 to 1.0 MJ kg^{-1} is likely sufficient to justify careful optimization of this parameter in actual practice. **(c)** The effect of CO_2 recycle ratio on energy requirements, where the CO_2-to-isobutanol feed ratio (by mass) has been fixed at 3:1 and the intermediate pressure fixed at 6.5 MPa. de Filippi and Moses reported extraction data obtained using a 0.6 recycle ratio for *n*-butanol[12] (literature). Note CO_2 recycle ratios greater than 0.87 cannot be achieved as the microbially-produced CO_2 from glucose conversion to isobutanol displaces approximately 13% (by mass) of the CO_2–recycle stream required for separation when the CO_2-to-isobutanol feed ratio is 3:1. Since the co-produced CO_2 is already at 10 MPa, this represents an additional energy benefit of the extraction-fermentation process, enhancing the overall efficiency of the process. Source data of Supplementary Figure 15B, C are provided as a Source Data file.

Supplementary Table 1. FACS analysis of SR7 expressing GFP using different promoters

	**Percent GFP positive (%)[a]**		**Median fluorescence (A.U.)[c]**	
**Sample**	– Inducer[b]	+ Inducer[b]	– Inducer	+ Inducer
pXyl empty	0.0 ± 0.0	0.0 ± 0.0	n/a[d]	n/a
pXyl *gfp*	40.5 ± 3.9	95.4 ± 1.9	608 ± 23	17,500 ± 2,600
pHysp empty	0.2 ± 0.2	0.0 ± 0.0	n/a	n/a
pHysp *gfp*	3.3 ± 0.2	97.4 ± 0.9	484 ± 12	8,900 ± 590
p43 empty	0.1 ± 0.1	–	n/a	–
p43 *gfp*	63.0 ± 3.9	–	1,860 ± 112	–

[a] GFP positive gate was established by gating above the autofluorescence observed for all empty plasmid containing controls, and members falling within this gate are reported as GFP positive. Average of three biological samples with standard deviation representing the error.

[b] Inducer for pXyl was 5 g l^{-1} xylose, for pHysp was 1 mM IPTG, and no inducer was added for p43 since it is a constitutive promoter.

[c] Median fluorescence for cells gated as GFP positive.

[d] n/a reported for any population where less than 1% was GFP positive.

Source data are provided as a Source Data file.

Supplementary Table 2. Colony forming units for SR7 pXyl *gfp* after 74 hours of culturing

	+ xylose cultures[a]			– xylose cultures[a]		
Sample	– Tet[b] (CFU/mL)	+ Tet[b] (CFU/mL)	Ratio[c] (+Tet/-Tet)	– Tet[b] (CFU/mL)	+ Tet[b] (CFU/mL)	Ratio[c] (+ Tet/– Tet)
# 1	3.05 E7	3.12 E7	1.02	3.91 E7	4.03 E7	1.03
# 2	2.90 E7	2.53 E7	0.87			
# 3	1.52 E7	1.55 E7	1.02			
**Average**			**0.97± 0.08**			

[a] SR7 pXyl *gfp* cells were grown in LB medium with 5 μg ml^{-1} tetracycline antibiotic. GFP production was induced at 2.5 hours for cultures (+ xylose) with 5 g l^{-1} xylose.
[b] Serial dilutions were used to determine the colony forming units (CFUs) for each SR7 culture. Samples were plated on LB agar plates with 5 μg ml^{-1} tetracycline (+ Tet) antibiotic to determine the number of cells that maintained the plasmid, and without tetracycline (– Tet) to determine total CFUs.
[c] Ratio of CFUs on the + Tet to – Tet plate, showing the percentage of cells still resistant to 5 μg ml^{-1} tetracycline. The average ratio of the + xylose cultures is shown along with the standard deviation as error.
Source data are provided as a Source Data file.

Supplementary Table 3. Growth frequency of SR7 strains grown under 10 MPa $scCO_2$ at 37 °C

		– xylose cultures[a]			+ xylose cultures[a]			**Total**		
Strain[b]	Plasmid size (kbp)	# samples	# growth[c]	% growth[d]	# samples	# growth[c]	% growth[d]	# samples	# growth[c]	% growth[d]
pXyl empty	7.4	8	2	25.0	29	7	24.1	37	9	24.3
pXyl *lacZ*	10.4	55	8	14.5	34	5	14.7	89	13	14.6
pXyl *kivD* $yqhD_{Ec}$[e]	10.1	27	7	25.9	90	26	28.9	117	33	28.2
**Total**		**90**	**17**	**18.9**	**153**	**38**	**24.8**	**243**	**55**	**22.6**
SR7 WT[f]		25	16	64.0						

[a]Cultures were grown in semi-defined medium with 4 g l^{-1} glucose, 0.05 g l^{-1} yeast extract, 100 mM L-alanine and 0.1x trace metal solution. For the induced cultures (+ xylose), 5 g l^{-1} xylose was also added at the beginning of the culturing time.
[b]SR7 cells containing a desired plasmid were prepared as endospores prior to culturing under $scCO_2$.
[c]Number of cultures that showed at least 10-fold cell growth as measured by fluorescence microscopy.
[d]Ratio of number of cultures showing growth to the total number of samples.
[e]The medium for all SR7 pXyl *kivD* $yqhD_{Ec}$ samples also contained 5 mM αKIV.
[f]Number of cultures showing growth for SR7 wild type that does not contain a plasmid grown under identical conditions as reported in Freedman, et al. (2018)[4].
Source data are provided as a Source Data file.

Supplementary Table 4. Alcohol production, glucose and αKIV consumption, and isobutanol yield for SR7 biofuel strains grown under 10 MPa $scCO_2$ at 37 °C

Sample	Xylose added[a] (g l^{-1})	Average/ Replicate[b]	Isobutanol titer (mM)[c]	Isopentanol titer (mM)[c]	Glucose consumed (mM)	Xylose consumed (mM)	αKIV consumed (mM)	Isobutanol yield on αKIV[d] (%)	Isobutanol yield on αKIV, glucose, and xylose[e] (%)
Low activity[f]									
pXyl empty	5	avg	0.00±0.00	0.00±0.00	0.28±0.11	0.07±0.07	0.73±0.40	0.0±0.0	0.0±0.0
pXyl *kivD* $yqhD_{Ec}$	0	avg	0.01±0.01	0.00±0.00	0.24±0.14	0.00±0.00	1.39±1.30	3.2±5.7	1.1±1.5
pXyl *kivD* $yqhD_{Ec}$	5	avg	0.04±0.07	0.01±0.01	0.19±0.20	0.31±0.48	0.64±0.53	3.0±3.6	2.2±2.7
High activity[f]									
pXyl empty	5	A	0.00	0.00	3.35	2.6	1.95	0	0
pXyl *kivD* $yqhD_{Ec}$	0	A	0.55	0.00	3.35	0.00	4.92	11	7
	0	B	0.66	0.11	1.09	0.00	3.43	19	15
pXyl *kivD* $yqhD_{Ec}$	5	A	1.14	0.34	3.21	0.94	3.12	37	16
	5	B	1.28	0.28	1.89	0.95	2.83	45	23
	5	C	1.26	0.32	1.30	0.83	2.79	45	26

[a] Cultures were grown in semi-defined medium with 4 g l^{-1} glucose, 0.05 g l^{-1} yeast extract, 100 mM L-alanine and 0.1x trace metal solution. For the induced cultures, 5 g l^{-1} xylose was also added at the beginning of the culturing time.

[b] For low activity cultures, an average value is reported for each set of samples, and error shown is the standard deviation (number of samples for each culture condition is provided in Supplementary Table 3).
For those samples that had high activity, data for each sample replicate (i.e. A) is provided.

[c] Aqueous phase titer.

[d] Isobutanol yield on αKIV was calculated for each sample by dividing the isobutanol titer (mM) by the concentration of αKIV consumed (mM). For low activity cultures, the average and standard deviation of all the samples in each set are reported, while individual replicates are shown for the high activity cultures.

[e] Isobutanol yield on αKIV, glucose and xylose was calculated for each sample by dividing the isobutanol titer (mM) by the sum of concentration of αKIV consumed (mM), concentration of glucose consumed (mM), and 5/6 of concentration of xylose consumed (mM). Converting 1 mole of glucose through glycolysis theoretically results in the production of 1 mole of isobutanol (loss of 2 CO_2). For xylose consumption, it was assumed that all the xylose entered glycolysis through various intermediates in the pentose phosphate pathway. Theoretically 6 moles of xylose are needed to produce 5 moles of isobutanol (loss of 10 CO_2), hence the 5/6 correction to the xylose consumption (mM).

[f] Low and high activity cultures are defined in Supplementary Figure 11. High activity cultures consumed at least 1 mM glucose and produced over 1 mM combined lactate and acetate.

Source data are provided as a Source Data file.

Supplementary Table 5. Isobutanol titer normalized to cell density for SR7 pXyl *kivD yqhD*$_{Ec}$ grown under 0.1 MPa CO_2 or 10 MPa scCO_2 with 5 g l^{-1} xylose and 5 mM αKIV[a]

Condition	Average/replicate[c]	Isobutanol titer (mM)	Cell count[d] ($\times 10^7$ cells ml^{-1})	Per-cell titer[e] ($\times 10^{-8}$ µmol $cell^{-1}$)
0.1 MPa CO_2	Avg[f]	4.43±0.03	20	2.21±0.00
10 MPa scCO_2[b]	A	1.14	5.14	2.23
	B	1.28	3.89	3.30
	C	1.26	3.33	3.79
	**Avg**	**1.23±0.08**	**4.12±0.93**	**3.11±0.80**

[a] Cultures were grown in semi-defined medium with 4 g l^{-1} glucose, 0.05 g l^{-1} yeast extract, 100 mM L-alanine, 0.1x trace metal solution, 5 g l^{-1} xylose, and 5 mM αKIV.
[b] Only samples with high activity (Supplementary Figure 11) are shown for 10 MPa scCO_2. Replicates are identical to those in Supplementary Table 4.
[c] For 0.1 MPa CO_2 cultures, an average value is reported, and error shown is the standard deviation. For high activity10 MPa scCO_2 cultures, data for each replicate is provided in addition to the average and standard deviation of the data (bottom row).
[d] Cell counts were measured by microscopy of syto9-stained cells collected on a filter.
[e] Per-cell isobutanol titer was found by dividing the isobutanol titer by the cell count.
[f] The average of the 0.1 MPa CO_2 samples was calculated using data from three independent cultures and the error shown is the standard deviation.
Source data are provided as a Source Data file.

Supplementary Table 6. scCO_2 process model parameters and energy requirements

	Atmospheric fermentation separate extraction	Full depressurization with no recycle	Partial depressurization with partial recycle	Partial depressurization with full recycle
Parameter				
Intermediate pressure (MPa)	n/a	0.1	6.5	6.5
CO_2 recycle ratio	0	0	0.5	0.87[a]
Inlet CO_2 flow rate (kg hr^{-1})	0.300	0.185	0.08	0.000
CO_2:isobutanol ratio[b]	3:1	3:1	3:1	3:1
Mass fraction isobutanol in product	0.970	0.960	0.972	0.973
Energy requirements				
Compressors (MJ kg^{-1})	4.25	2.20	0.89	0.15
Heat exchangers (MJ kg^{-1})	1.12	1.14	0.74	0.49
Liquid pump (MJ kg^{-1})	0.85	0.74	0.75	0.75
Separators and turbines (MJ kg^{-1})	0.54	0.27	0.04	0.06
**Total (MJ kg^{-1})**	**6.76**	**4.35**	**2.43**	**1.44**

[a] CO_2 recycle ratios greater than 0.87 cannot be achieved as the microbial-produced CO_2 from glucose conversion to isobutanol displaces approximately 13% (by mass) of the CO_2-recycle stream required for separation when the CO_2-to-product ratio is 3:1. Since the co-produced CO_2 is already at 10 MPa, this represents an additional energy benefit of the extraction-fermentation process.
[b] Calculated by mass.
Source data are provided as a Source Data file.

Supplementary Table 7: Strains and plasmids

Strains

Name	Description	Reference/Source
Bacillus megaterium		
SR7[a]	scCO_2-tolerant, wild type strain	Freedman A.J.E., et al. 2018[4]
E. coli		
DH5α	F^- φ80*lacZ* ΔM15 Δ(*lacZYA argF*) U169 *deoR recA1 endA1 hsdR17*(r_k^-, m_k^+) *phoA supE44 thi*-1 *gyrA96 relA1* λ^-	Invitrogen

[a] The genome sequence of SR7 has been deposited to NCBI under GenBank accession numbers CP022674-CP022679.

Plasmids

Name	Description[a]	Reference/Source
pRBBm34	Shuttle vector for cloning in *E. coli* (Amp^r) and expression in *B. megaterium* (Tet^r); P_{xylA}–GFP+	Addgene (#48114)
pDR111	Vector for cloning in *E. coli* (Amp^r) and integration in *B. subtilis* at *amyE* locus ($Spec^r$); $P_{hyperspank}$	Ben-Yehuda, et al. 2003[14]
pXyl empty	P_{xylA}; *xylR* (Amp^r:*E.coli* ,Tet^r:*B. megaterium*)	This work
pXyl *gfp*	P_{xylA}–gfp_{SF}; *xylR* (Amp^r, Tet^r)	This work
pHysp empty	$P_{hyperspank}$; *lacI* (Amp^r, Tet^r)	This work
pHysp *gfp*	$P_{hyperspank}$–gfp_{SF}; *lacI* (Amp^r, Tet^r)	This work
p43 empty	P_{43} (Amp^r, Tet^r)	This work
p43 *gfp*	P_{43}–gfp_{SF} (Amp^r, Tet^r)	This work
pXyl *lacZ*	P_{xylA}–$lacZ_{Ec}$; *xylR* (Amp^r, Tet^r)	This work
pXyl *kivD*	P_{xylA}–$kivD_{Ll}$; *xylR* (Amp^r, Tet^r)	This work
pXyl *kivD* $ADH6_{Sc}$	P_{xylA}–$kivD_{Ll}$–$ADH6_{Sc}$; *xylR* (Amp^r, Tet^r)	This work
pXyl *kivD* $adhA_{Ll}$	P_{xylA}–$kivD_{Ll}$–$adhA_{Ll}$; *xylR* (Amp^r, Tet^r)	This work
pXyl *kivD* $adhP_{Ec}$	P_{xylA}–$kivD_{Ll}$–$adhP_{Ec}$; *xylR* (Amp^r, Tet^r)	This work
pXyl *kivD* $yqhD_{Ec}$	P_{xylA}–$kivD_{Ll}$–$yqhD_{Ec}$; *xylR* (Amp^r, Tet^r)	This work
pXyl *kivD* $adhA_{Bm}$	P_{xylA}–$kivD_{Ll}$–$adhA_{BmSR7}$; *xylR* (Amp^r, Tet^r)	This work

[a] Abbreviations: Amp^r–ampicillin resistance marker, Tet^r–tetracycline resistance marker, gfp_{SF}– SuperFolder GFP, *Ec*– *E. coli*, *Ll*– *L. lactis*, *Sc*– *S. cerevisiae*, *BmSR7*– *B. megaterium* strain SR7.

Supplementary Table 8. Oligonucleotide primers

Name	Sequence (5'–3')[a]
pXyl seq for	tcctttgtttatccaccgaactaag
pXyl seq rev	GTTCTGCCAAGGGTTGGTTTG
pHysp seq for	ctacaaggtgtggcataatgtgtg
pHysp seq rev	GTACGTACGATCTTTCAGCCGAC
p43 seq for (pRBBm34 rev)	gcactgtccgaccgctttgg
27F (16s for)	agagtttgatcmtggctcag
1492R (16s rev)	GGTTACCTTGTTACGACTT
$xylE_{Bm}$ seq for	cattggaagagtgtgcaaacg
$xylE_{Bm}$ seq rev	GCTATCCACTTTCTTCATAAAGGTTCC
gfp seq rev	TACCCGAGGTCTCGtacttatagagttcatccatgccatg
RepU seq for	catcacttatacaacaatatggcccg
RepU seq rev	GGTTGCACAGAAAAACCCCATCTG
SpeI gfp for	gtccaaactagtagcaagggcgaagagctttttacc
gfp SphI rev	TAGCTTCCCGGGGCATGCTCATTACTTATAGAGTTCATCCATGCCATGAG
pRBBm34 for	cggcggcacctcgctaac
pRBBm34-pHysp for	cacacccgtcctgtggatctgactctctagcttgaggcatc
lacI-pRBBm34 rev	GTTAGCGAGGTGCCGCCGGGATCCTAACTCACATTAATTGCG
bla for	ccaatgcttaatcagtgaggcacc
bla rev	GGTGCCTCACTGATTAAGCATTGG
RBS-start for	agggggaaatgtacaatggtccaaactag
pRBBm34-p43 for	cacacccgtcctgtggatctgtgtcgacgtgcatgcagg
p43-RBS-start rev	GTTTGGACCATTGTACATTTCCCCCTTATAATGGTACCGCTATCACTTTATATTTTAC
SalI RBS *gfp* for	agcttagtcgacagggggaaatgtacaatgagcaagggcgaagagc
gfp NheI rev	GCATGCGGCTAGCTCATTACTTATAGAGTTCATCCATGCCATGAG
SpeI $lacZ_{Ec}$ for	gtccaaactagtaccatgattacggattcactggc
$lacZ_{Ec}$ *SphI* rev	CCGCCGGCATGCTCATTATTTTTGACACCAGACCAACTGG
SpeI $kivD_{Ll}$ for	gtccaaactagtatgtatacagtaggagattacctattagaccg
$kivD_{Ll}$ *BamHI SphI* rev	GAGGAGCATGCGAGCTCGGATCCTCATTATGATTTATTTTGTTCAGCAAATAGTTTACCC
SalI RBS $kivD_{Ll}$ *for*	agcttagtcgacagggggaaatgtacaatgatgtatacagtaggagattacctattagac
$kivD_{Ll}$ *NheI* rev	CATGCGGCTAGCTCCCGGGTACTAGTTCATTATGATTTATTTTGTTCAGCAAATAG
BamHI RBS $ADH6_{Sc}$ for	gaggagggatcctcgacagggggaaatgtacaatgagctacccggaaaagttcg
$ADH6_{Sc}$ *SphI rev*	CCGCCGGCATGCAATGCGGCCGCTCATTAGTCGCTGAATTCTTTATCGTAACCAACC
BamHI $adhA_{Ll}$ for	taatgaggatcctcgacagggggaaatgtacaatgaaagcagcagtagtaagacacaatc
$adhA_{Ll}$ *SphI* rev	GCATGCAATGCGGCCGCTCATTATTTAGTAAAATCAATGACCATTCGGCC
BamHI $adhP_{Ec}$ for	taatgaggatcctcgacagggggaaatgtacaatgaaggctgcagttgttacgaag
$adhP_{Ec}$ *SphI* rev	GCATGCAATGCGGCCGCTCATTAGTGACGGAAATCAATCACCATGC
BamHI $yqhD_{Ec}$ for	taatgaggatcctcgacagggggaaatgtacaaatgaacaactttaatctgcacaccc
$yqhD_{Ec}$ *SphI* rev	GCATGCAATGCGGCCGCTCATTAGCGGGCGGCTTCGTATATAC
BamHI $adhA_{Bm}$ for	aatgaggatcctcgacagggggaaatgtacaatgtgtaataatcatcaacatacaacaac
$adhA_{Bm}$ *SphI* rev	GCATGCAATGCGGCCGCTCATTACAGTGTAGAAATGTCAATAACAAATCG

[a] Primers listed in lowercase are forward primers. Primers listed in uppercase are reverse primers.

Supplementary References

1. Biedendieck R, *et al.* Systems biology of recombinant protein production using *Bacillus megaterium*. *Methods Enzymol* **500**, 165-195 (2011).

2. Moro A, Sánchez JC, Serguera C. Transformation of *Bacillus megaterium* by electroporation. *Biotechnology Techniques* **9**, 589-590 (1995).

3. Richhardt J, Larsen M, Meinhardt F. An improved transconjugation protocol for *Bacillus megaterium* facilitating a direct genetic knockout. *Appl Microbiol Biotechnol* **86**, 1959-1965 (2010).

4. Freedman AJ, Peet KC, Boock JT, Penn K, Prather KL, Thompson JR. Isolation, Development, and Genomic Analysis of *Bacillus megaterium* SR7 for Growth and Metabolite Production Under Supercritical Carbon Dioxide. *Frontiers in Microbiology* **9**, (2018).

5. Tompsett GA, *et al.* Extraction Rate and Energy Efficiency of Supercritical Carbon Dioxide Recovery of Higher Alcohols from Dilute Aqueous Solution. *Energy Technology* **6**, 683-693 (2018).

6. Stockhardt JS, Hull CM. Vapor-Liquid Equilibria and Boiling-Point Composition Relations for Systems *n*-Butanol–Water and Isobutanol–Water. *Industrial & Engineering Chemistry* **23**, 1438-1440 (1931).

7. Nielsen DR, Leonard E, Yoon SH, Tseng HC, Yuan C, Prather KL. Engineering alternative butanol production platforms in heterologous bacteria. *Metab Eng* **11**, 262-273 (2009).

8. Atsumi S, Hanai T, Liao JC. Non-fermentative pathways for synthesis of branched-chain higher alcohols as biofuels. *Nature* **451**, 86-89 (2008).

9. Panagiotopoulos AZ, Reid RC. Multiphase high pressure equilibria in ternary aqueous systems. *Fluid Phase Equilibria* **29**, 525-534 (1986).

10. Chen H-I, Chang H-Y, Chen P-H. High-Pressure Phase Equilibria of Carbon Dioxide + 1-Butanol, and Carbon Dioxide + Water + 1-Butanol Systems. *Journal of Chemical & Engineering Data* **47**, 776-780 (2002).

11. Laitinen A, Kaunisto J. Supercritical fluid extraction of 1-butanol from aqueous solutions. *The Journal of Supercritical Fluids* **15**, 245-252 (1999).

12. de Filippi RP, Moses JM. Extraction of organics from aqueous solutions using critical-fluid carbon dioxide. *Biotechnology and Bioengineering Symposium* **12**, 205-219 (1982).

13. Oudshoorn A.Recovery of bio-based butanol. *Thesis*. TU Delft. Biotechnology Department. (2012). ISBN: 9789053355619.

14. Ben-Yehuda S*, et al.* Defining a centromere-like element in *Bacillus subtilis* by Identifying the binding sites for the chromosome-anchoring protein RacA. *Mol Cell* **17**, 773-782 (2005).
